# Effects of Lifestyle Interventions on the Improvement of Chronic Non-Specific Low Back Pain: A Systematic Review and Network Meta-Analysis

**DOI:** 10.3390/healthcare12050505

**Published:** 2024-02-20

**Authors:** Pablo Herrero, Paula Val, Diego Lapuente-Hernández, Juan Nicolás Cuenca-Zaldívar, Sandra Calvo, Eva María Gómez-Trullén

**Affiliations:** 1 Department of Physiatry and Nursing, Faculty of Health Sciences, University of Zaragoza, 50009 Zaragoza, Spain; pherrero@unizar.es (P.H.); paulavc1206@gmail.com (P.V.); d.lapuente@unizar.es (D.L.-H.); evagomez@unizar.es (E.M.G.-T.); 2 iHealthy Research Group, University of Zaragoza, IIS Aragon, 50009 Zaragoza, Spain; 3 Grupo de Investigación en Fisioterapia y Dolor, Departamento de Enfermería y Fisioterapia, Facultad de Medicina y Ciencias de la Salud, Universidad de Alcalá, 28801 Alcalá de Henares, Spain; nicolas.cuenca@salud.madrid.org; 4 Research Group in Nursing and Health Care, Puerta de Hierro Health Research Institute–Segovia de Arana (IDIPHISA), 28222 Madrid, Spain; 5 Primary Health Center “El Abajón”, 28231 Las Rozas de Madrid, Spain

**Keywords:** low back pain, lifestyle, sedentary behavior, pain intensity, functional disability, multivariate network meta-analysis, non-specific low back pain

## Abstract

Chronic non-specific low back pain (CNSLBP) is a highly prevalent musculoskeletal condition that has a great socioeconomic impact on health systems. Instead of focusing on mechanical causes and direct workload in the development of CNSLBP, genetics, psychosocial environment, lifestyle and quality of life are coming to the forefront in its approach. The main objective was to analyze whether interventions aimed at modifying lifestyle can be effective in improving pain intensity and functional disability in CNSLBP. A search in PubMed, Web of Science, Scopus and SportDiscus databases was performed. Both a univariate and a multivariate network meta-analysis were applied with the difference pre/post-treatment. A total of 20 studies were included for qualitative analysis, of which 16 were randomized clinical trials with a moderate–high methodological quality and were part of the quantitative analysis. The interventions that had the greatest effect in reducing pain intensity were cognitive therapy combined with functional exercise programs, lumbar stabilization exercise and resistance exercise; meanwhile, for functional disability, they were functional exercise programs, aerobic exercise and standard care. In conclusion, a multimodal intervention aimed at changing one’s lifestyle that encompasses cognitive, behavioral, and physical aspects seems to be highly effective in improving pain intensity and functional disability caused by CNSLBP; however, it is not yet known if these improvements are maintained in the long term.

## 1. Introduction

Low back pain (LBP) is the most common musculoskeletal condition worldwide, experienced by 70–80% of the adult population at some point in life, and it is considered the main cause of work absenteeism, years lived with functional disability and one of the first five diagnoses established in Primary Care consultations [1,2]. In addition, the ageing and gradual growth of the population are contributing to an increase in the number of people with disabilities related to LBP, which generates a significant socioeconomic impact on healthcare systems [3,4]. However, in 85–95% of cases, its specific etiology is not clear, and therefore, it is known as non-specific (NSLBP) [5,6]. Most of the episodes of NSLBP improve significantly in the first 6 weeks, although up to 40% seem to experience symptoms beyond 3 months, which is classified as chronic (CNSLBP) [7].

A better understanding of the clinical course of the CNSLBP is needed since there is a lack of knowledge locally in terms of investigating the incidence of LBP and its related risk factors, with a clear understanding of its related disability among patients. A personalized approach to rehabilitation planning that takes into account patient-specific risk factors and occupational and environmental components involving the multidimensional perspective that is CNSLBP is needed [8,9,10,11].

In 2018, a series of studies in The Lancet insisted on prioritizing an improvement in the care’s quality for the surgical and non-surgical management of LBP [4,12,13], since providing guideline-compliant treatment as part of a routine clinical practice continues to be a challenge for physical therapists [14,15]. Recently, clinical practice guidelines for LBP [16,17,18] have reduced the emphasis on surgical interventions and pharmacological treatments, such as opioids [17,18], encouraging non-pharmacological interventions as first-choice treatments for CNSLBP, with the aim of empowering the individual with LBP to self-manage [16,17] and promoting active interventions for pain relief, functional improvement and/or reduction in functional disability [18]. Many of these interventions are related to changes in lifestyles, promoting strategies that address psychosocial factors and focus on improving function, counseling, education or physical activity programs [16,17].

Despite the high prevalence of people with CNSLBP and the recommendations for active interventions in clinical practice, to our knowledge, there are no reviews that have compared the effectiveness of different interventions aimed at modifying lifestyle for CNSLBP. Therefore, the aim of this systematic review and network meta-analysis was to examine which lifestyle interventions could lead to better improvements in pain intensity and functional disability in people suffering from CNSLBP.

## 2. Materials and Methods

The systematic review and meta-analysis were conducted following the standard protocol outlined in the Preferred Reporting Items for Systematic Reviews and Meta-Analyses (PRISMA; Supplementary Table S1) [19]. Additionally, the study was registered with PROSPERO, the international prospective register of systematic reviews (reference number CRD42022315090).

### 2.1. Eligibility Criteria

The inclusion and exclusion criteria for the study were determined using the PRISMA checklist and the PICOS formula (participants, interventions, comparators, outcomes and study design) [19].

The following criteria were applied to include studies: (1) participants were adults (>18 years old) suffering from CNSLBP for at least 3 months, with no restrictions based on race or gender; (2) studies in which patients underwent any lifestyle intervention, including health education, were considered; (3) studies comparing groups of CNSLBP receiving different interventions or where there was a control group with no intervention; (4) all types of clinical trials, such as randomized controlled trials (RCTs), matched-controls and cohorts, among others, considering only peer-reviewed journal articles; (5) outcomes related to both pain intensity and functional disability; and (6) studies written in either English or Spanish.

Conversely, the exclusion criteria for studies were as follows: (1) participants with specifics causes of low back pain (e.g., spinal canal stenosis or herniated disc) or severe psychiatric, neurological, infectious, oncological, renal or inflammatory conditions, i.e., conditions necessitating alternative treatments or hindering participation in the interventions); (2) studies whose lifestyle interventions are based solely on nutrition or psychological therapy; (3) non-human research or animal testing; (4) alternative forms of publications, including book sections, letters to the editor, conference abstracts or generic material, protocols, reviews or meta-analysis; and (5) information not disseminated in peer-reviewed journals or limited to abstracts only.

### 2.2. Data Sources and Search

An electronic search was conducted and ended on 19 March 2022. PubMed, Web of Science, Scopus and SportDiscus databases were evaluated and consulted to identify studies. Regarding the search terms, two categories were defined: the first related to population (“low back pain”) and the second to interventions (“lifestyle” and “sedentary behavior”). The chosen search terms resulted from an initial exploration of the literature and the identification of keywords. After determining these terms, they were entered into various databases’ search engines, where they were combined using the Boolean operators “AND” and “OR”. The complete search strategy was as follows: low back pain AND (lifestyle OR sedentary behavior).

### 2.3. Study Selection

To assess compliance with the inclusion criteria, two reviewers (P.V. and C.G.-A.) independently evaluated each study. To mitigate bias, they followed a standardized methodology established after reaching an agreement on the execution of search equations. Subsequently, the outcomes of their individual reviews were compared. Initially, all records were extracted from the three databases and imported into the bibliographic management tool “Mendeley version 1.19.8” to identify and eliminate duplicate publications. The first screening involved a preliminary assessment of articles based on information available in the title and abstract, selecting those that potentially met the inclusion criteria. A subsequent phase of screening involved a thorough examination of the full texts of the surviving studies from the previous phase. Studies meeting all inclusion criteria were then chosen. The selected studies underwent a comparative analysis, and in instances of disagreement, a third researcher (E.G.-T.) was consulted to facilitate consensus.

### 2.4. Data Extraction Process

Two evaluators (P.V. and C.G.-A.) conducted individual data collection from studies and subsequently cross-referenced the extracted data to ensure consistency. In cases of disagreement between the data extractors, a resolution was achieved by consulting a third party (E.G.-T.).

The data extraction process included retrieving the following information from each study: author and publication year, sample characteristics (including sample size, gender, and age), intervention details, comparator/control group specifications, main outcomes (pertaining to pain intensity and functional disability), additional self-reported outcomes (covering physical activity, functionality, sleep quality and/or psychosocial factors) and main findings.

### 2.5. Assessment of Methodological Quality and Risk of Bias

Four distinct tools were employed to evaluate the methodological quality and risk of bias in the studies included, tailored to their respective study designs. The two reviewers, P.V. and C.G.-A., conducted their assessments independently, and the results were subsequently compared, excluding the involvement of a third reviewer.

For RCTs and studies employing random assignment, the PEDro scale and the Cochrane Risk of Bias 2.0 (RoB2) tool were utilized. The PEDro scale, consisting of 11 items, assigns a score of 1 if the article meets the criteria and 0 if it does not. The items assess external validity (Item 1), internal validity related to study design (Items 2–9) and interpretability of results (Items 10 and 11) [20,21]. The maximum achievable score is 10 points, with the first item not considered in the final score. Interpretation of the score-categorized articles scoring at least 6 out of 10 as “high quality”, those scoring between 4 and 5 as “moderate quality” and articles with less than 4 points as “low quality” [20,21]. Risk of bias for RCTs was evaluated using the Cochrane Risk of Bias 2.0 (RoB2) tool, focusing on “allocation to intervention” across five domains: (1) randomization process, (2) deviations from planned interventions, (3) missing outcome data, (4) outcome measurement, and (5) selection of reported outcome [22]. The overall risk of bias was categorized as either “low risk”, “some concerns” or “high risk” for each outcome [22].

For studies involving non-randomized interventions, the Risk of Bias in Non-Randomized Studies of Interventions (ROBINS-I) tool [23] was employed. ROBINS-I assesses the risk of bias across seven domains: (1) confusion, (2) selection of study participants, (3) classification of interventions, (4) deviations from previously stipulated interventions, (5) lack of data or information, (6) measurement of the variables and (7) selection of the exposed results. Each domain was scored as “low risk”, “moderate risk”, “serious risk”, “critical risk” or “no information”. The study’s total score was then determined based on the evaluation of each domain.

Finally, for observational studies, the National Heart, Lung and Blood Institute (NHLBI) Quality Assessment Tool for Observational Cohorts and Cross-Sectional Studies was used [24]. The quality of the studies was assessed by answering a series of questions scoring as “good”, “fair” or “poor”.

### 2.6. Statistical Analysis

For the statistical analysis, the program R Ver. 4.1.3 (R Foundation for Statistical Computing, Institute for Statistics and Mathematics, Welthandelsplatz 1, 1020 Vienna, Austria) was used. The packages metafor [25] for multivariate analysis and netmeta [26] for univariate analyses were used.

In the articles in which the means and standard deviations were not reported, these were requested from the corresponding authors by e-mail. When it was not possible to obtain a response, in the articles in which the results were shown with median and interquartile range, these were transformed into mean and standard deviation, using the appropriate formulas [27,28]; and in the articles in which the intervals of confidence were given instead of the standard deviation, this was calculated using the appropriate formulas [29]. Finally, in the articles that did not report any data, these were extracted from the graphs, using the WebPlotDigitizer Ver. 4.6 [30] program.

In the article by Bendix et al. [31], the medians provided by the author were used in the pre-treatment values; since the interquartile ranges were not reported, they could not be calculated. For this same reason, the pre-treatment standard deviations of this study were imputed using the predictive mean matching. Finally, data from the two functional exercise program groups were combined using the appropriate formulas [29].

A frequentist network meta-analysis (NMA) was performed with the pre/post-treatment difference. This was calculated with the appropriate formulas when the studies did not report it [29] and by assigning a value of 0.7 to the pre/post-treatment correlation coefficient to obtain a conservative estimate [32], as was performed in other studies [33,34,35,36,37].

A multivariate NMA was applied combining the pain intensity and functional disability outcomes, using the standardized mean difference (SMD), for which the correlations between the Visual Analog Scale (VAS) and Numeric Rating Scale (NRS) of pain intensity and the Oswestry Disability Index (ODI) of functional disability were used, reported by Bielewicz et al. [38]; and Roland Morris Disability Questionnaire (RMDQ) of functional disability, reported by Kersten et al. [39]. Two univariate NMAs were also carried out for pain intensity, using the mean difference (MD); and for functional disability, using the SMD. The objective of this analysis was to evaluate the effectiveness of the treatments on pain intensity and functional disability in combination, and on each of them separately. In both the multivariate and univariate NMAs with the functional disability outcome, SMD was used as both include different scales, while for the univariate NMA with the pain intensity outcome, MD was used, as all studies used the same scale.

The selection of a fixed- or random-effects model was carried out in the multivariate NMA, using the Likelihood Ratio Test (LRT) between both, evaluating the level of significance and the values of the Akaike Information Criterion (AIC) and the Bayesian Information Criterion (BIC). In the case of the univariate NMA, the value and level of significance of the inconsistency between studies were compared using the Cochrane test [40].

The assessment of transitivity was appraised under the assumption that all examined interventions yield consistent results, irrespective of the specific study they are associated with. To ensure this, it was verified that the confounding variables, such as age and male/female ratio, exhibited comparable distribution across all comparisons. This verification was accomplished by employing a network structure graph, where the size of the nodes was weighted by the covariates. Visual inspection was then conducted to identify any imbalances in the comparisons [41].

In all NMAs, heterogeneity was assessed by estimating the between-study variance, τ^2^, calculated with the REML (Restricted Maximum Likelihood) estimator, with the total and disaggregated intra- and between-study Cochrane Q test, as well as with the estimator I^2^ being defined with the latter as follows: 0–30%, non-important heterogeneity; 30–50%, moderate heterogeneity; 50–75%, large heterogeneity; and 75–100%, important heterogeneity.

In the case of univariate NMAs, the net-splitting method was used to assess consistency, analyzing the significance level of the Z statistic to detect disagreement between the direct and indirect comparisons of each intervention, as well as by evaluating the value of the mean length of connections in both direct and indirect comparisons, taking values greater than 2 as the cutoff point. In both the univariate and multivariate NMAs, the contribution of each study to global inconsistency was assessed using the matrix of contributions in the first case and evaluating the contribution to the value of the Cochrane test for the heterogeneity of each study in the second.

The effectiveness of the treatments was analyzed using the league table for direct comparisons, ranking of P-score and SUCRA (Surface Under the Cumulative Ranking Curve) and visual inspection of the rankogram.

Finally, publication bias was analyzed using the adjusted funnel plot for each comparison, as well as the Egger, Begg–Mazumdar and Thompson–Sharp tests.

## 3. Results

### 3.1. Study Selection

**Figure 1:**
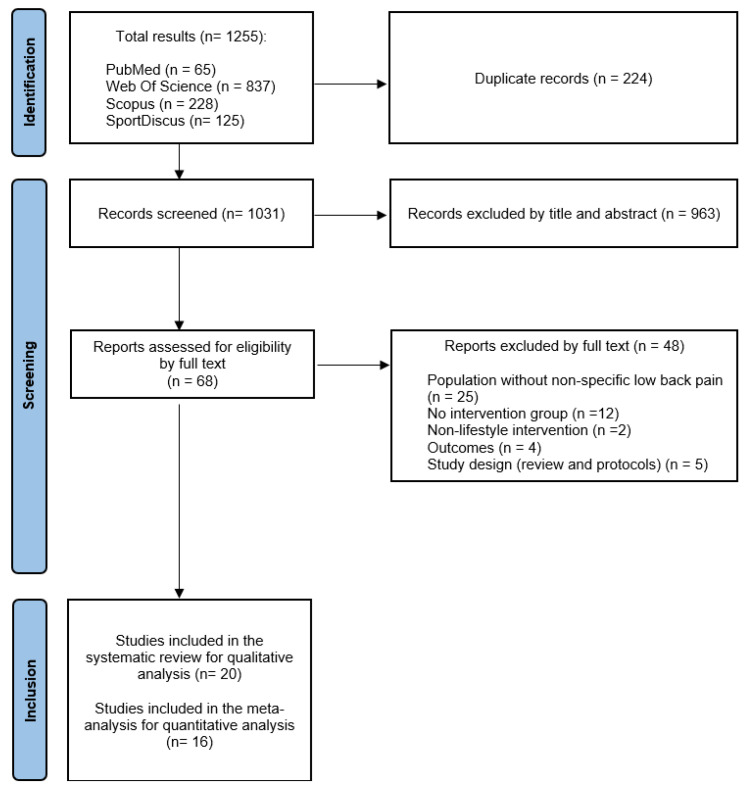
PRISMA flow diagram.

Following the implementation of the strategies outlined earlier, a cumulative total of 1255 studies were identified across the four databases (PubMed, 65; Web of Science, 837; Scopus, 228; and SportDiscus, 125). After eliminating duplicate studies through the use of the Mendeley bibliographic manager, 1031 articles were chosen for further examination. Initial screening, involving the review of titles and abstracts, resulted in a narrowed selection of 68 studies. Subsequently, during the second screening, these studies were thoroughly assessed in full text, leading to the exclusion of 48 studies that did not meet the inclusion criteria. Consequently, 20 articles were deemed suitable for qualitative analysis, with 16 of them being RCTs and, as such, included in the quantitative analysis. The flow diagram (Figure 1) provides a detailed depiction of the study search and selection process, along with the various reasons for exclusion, following the PRISMA criteria.

### 3.2. Study Characteristics

The data extracted from the 20 articles are presented in Table 1, arranged alphabetically by the last name of the first author. All the studies selected were RCTs, except for three non-randomized controlled intervention studies [42,43,44] and one prospective observational study [45].

#### 3.2.1. Sample

The samples of the selected studies range from 27 participants in the study by Kell et al. [46] to 650 in the study by Sofi et al. [42]. A total of 2525 participants were included in this review, of whom 909 were men (36.0%) and 1564 were women (61.9%), regardless of the study by Khodadad et al. [47], in which the gender of the participants was not specified.

Regarding the characteristics of the participants, all of them present CNSLBP (at least 3 months of evolution). The mean age of the participants was 46.4 ± 7.7 years. Body Mass Index was reported in 55% of the studies, with a mean of 26.9 ± 3.0 kg/m^2^. The mean duration of pain was only reported in the studies by Cuesta-Vargas et al. [48], Járomi et al. [49] and Kell et al. [46], with the mean of these samples being 59.4 ± 48.0 weeks. In addition, it is noteworthy that several studies included subjects with sedentary lifestyles, overweight/obesity (Body Mass Index = 25–40 kg/m^2^) and with professions that require prolonged sitting times (office workers).

#### 3.2.2. Intervention and Follow-Up

Regarding the applied interventions, 40% of studies compared an intervention related to lifestyle and a control group that that received no treatment or only advice and recommendations [31,42,44,45,49,50,51,52], 35% of studies compared two groups with different interventions [43,48,53,54,55,56,57,58] and the remaining 25% compared three groups (two interventions and one control) [31,46,47,59,60].

The duration of the intervention varied from 1 week [57,58] to 12 months [42,56], with the most frequent being 3 months, as was the case in 30% of the studies [43,49,54,55,59,60].

In 45% of the studies [31,50,51,52,53,54,55,56,59], a follow-up was introduced, either during the intervention or time after it had finished.

#### 3.2.3. Outcomes

In total, 60% of the studies evaluated both pain intensity and functional disability outcomes [43,44,48,49,50,51,52,53,54,55,56,59]. In addition, 70% of the studies evaluated other outcomes, such as quality of life, physical activity, functionality, sleep quality and psychosocial aspects (stress, fear-avoidance, self-efficacy, and anxiety or depression, among others) [31,42,44,45,46,47,48,50,52,53,54,55,56,58].

Pain intensity was evaluated in 50% of studies, using the numerical VAS [42,43,44,46,47,48,49,50,51,60], and in 40% of them, using a NRS [31,52,53,54,55,56,58,59], both with 11 points (0–10), with 0 being the absence of pain and 10 unbearable pain [61].

Functional disability was measured in 40% of studies [31,44,45,46,47,54,55,57] with the ODI, with 10 questions, each evaluated between 0 and 5 points [62]. In total, 35% of the studies [45,48,51,52,53,56,59] were evaluated with the RMDQ, made up of 24 questions [62]. Only one study [43] used the Quebec Low Back Pain Rating Scale (QLBPRS), which contains 20 questions [63].

### 3.3. Methodological Quality and Risk of Bias

**Figure 2:**
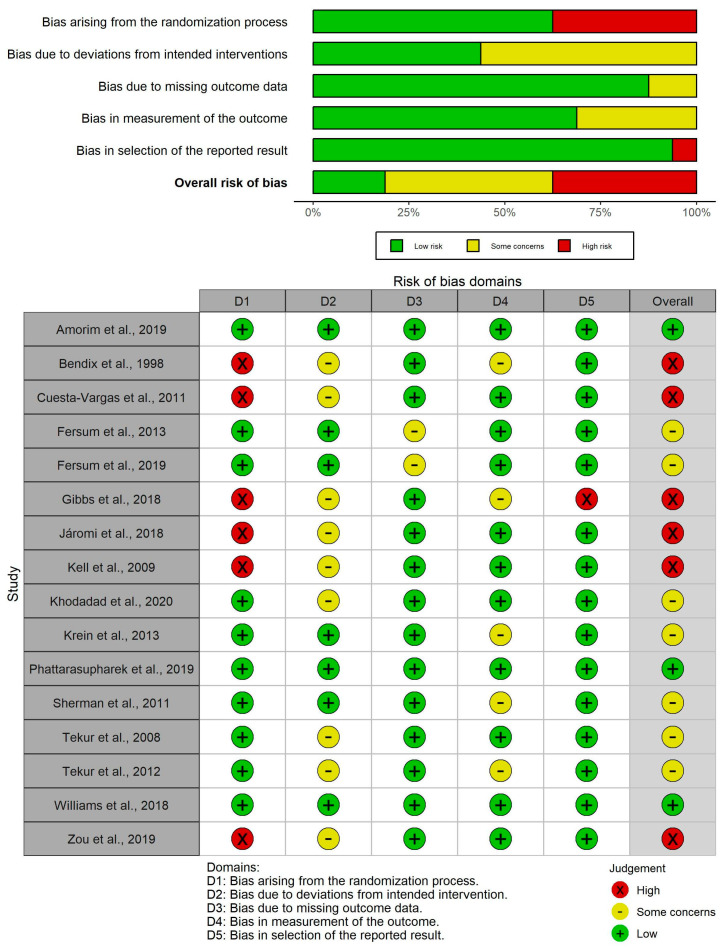
RoB2 risk of bias plots [31,46,47,48,49,50,51,52,53,54,55,56,57,58,59,60].

A total of sixteen RCTs were evaluated with the PEDro scale and the RoB2 tool. On the one hand, methodological quality scores ranged from 4 to 8, which means that all studies are considered to be between moderate and high quality. The average quality of all studies analyzed using the PEDro scale was 6.5; 14 studies obtained a score between 6 and 8 (“high quality”) [46,47,48,49,51,52,53,54,55,56,57,58,59,60], whereas the remaining 2 studies received a score of 4 or 5 (“moderate quality”) [31,50] (Table 2). On the other hand, the RoB2 tool showed that the overall outcome in terms of risk of bias varied from low risk in three studies [51,52,53] to high risk in six studies [31,46,48,49,50,60], with some concerns for the remaining seven studies [47,54,55,56,57,58,59] (Figure 2).

**Table 1:** Characteristics of included studies.

Author and Year	Study Design	Sample Characteristics	Intervention	Comparator/ Control	Main Outcomes	Other Outcomes	Follow-Up	Main Results
Amorim et al., 2019 [53]	RCT	IG: *n* = 34 (15 F, 19 M); Age = 59.5 ± 11.9; BMI = 28.9 ± 6.0 CG: *n* = 34 (19 F, 15 M); Age = 57.1 ± 14.9; BMI = 27.2 ± 5.1	Promotion of physical activity (information on sedentary behavior + personalized physical activity plan with coaching + motivational interviews + telephone calls)	Promotion of physical activity (information without follow-up)	Pain intensity (NRS) Disability (RMDQ)	Physical activity (IPAQ); fear avoidance (FABQ); depression and anxiety (DASS); sleep quality (PSQI)	Baseline and 6 months (follow-up every week)	Non-significant 1% weekly reduction in RMDQ (*p* = 0.66) No between-group SSD in NRS or RMDQ (*p* = 0.815 and *p* = 0.722, respectively)
Baena-Beato et al., 2013 [44]	Non-randomized controlled clinical trial	IG: *n* = 21 (12 F, 9 M); Age = 50.9 ± 9.6 GC: *n* = 17 (10 F, 7 M); Age = 46.2 ± 9.8	Aquatic therapy (resistance + aerobic + mobility exercise)	Waiting list + recommendations on ergonomics, healthy lifestyle and exercise	Pain intensity (VAS) Disability (ODI)	Quality of life (SF-36); Functionality (functional tests)	Baseline and 2 months	Between-group SSD in favor of IG in VAS and ODI (*p* < 0.001)
Bendix et al., 1998 (Project A) [31]	RCT	IG (A1): *n* = 50 (35 F; 15 M); Age = 41 CG (A2): *n* = 49 (36 F; 13 M); Age = 41	A1: Functional restoration (intensive physical exercise + psychological pain management + patient education)	A2: No intervention	Pain intensity (NRS)	Ability to work; ADLs; sports activity	Baseline, 3 weeks and 2 years	No between-group SSD in NRS (*p* = 0.5)
Bendix et al., 1998 (Project B) [31]	RCT	IG 1 (B2): *n* = 28 (21 F; 7 M); Age = 42.6 IG 2 (B3): *n* = 34 (25 F; 9 M); Age = 42.6 CG (B1): *n* = 40 (29 F; 11 M); Age = 38.9	B2: Intensive physical training + patient education B3: Intensive physical training + psychological pain management	B1: Same as A1	Same as Project A	Same as Project A	Baseline, 6 weeks and 2 years	Between-group SSD only when comparing B1 vs. B3 in favor of B1 (*p* = 0.003)
Cuesta-Vargas, 2011 [48]	RCT	IG: *n* = 25 (13 F, 12 M); Age = 39.8 ± 11.2; BMI = 26.2 ± 3.9; PD: 14.3 ± 9.4 weeks CG: *n* = 24 (14 F, 10 M); Age = 37.6 ± 13.2; BMI = 25.2 ± 4.5; PD: 16.9 ± 9.5 weeks	Multimodal program + Aquatic aerobic exercise	Multimodal program (therapeutic exercise + manual therapy + education)	Pain intensity (VAS) Disability (RMDQ)	Quality of life (SF-12); Functionality (functional tests)	Baseline and 15 weeks	Intragroup SSD for both groups in VAS and RMDQ (IG: *p* < 0.001 and *p* < 0.01, respectively; CG: *p* < 0.001) Between-groups SSD in favor of the IG in VAS (*p* < 0.05)
Fersum et al., 2013 [54]	RCT	IG: *n* = 51 (27 F, 24 M); Age = 41.0 ± 10.3; BMI = 25.6 ± 4.0 CG: *n* = 43 (21 F, 22 M); Age = 42.9 ± 12.5; BMI = 25.2 ± 3.5	Cognitive–functional therapy (cognitive component, specific movement exercises, integration of ADLs and physical activity program)	Multimodal program (manual therapy + general therapeutic exercise)	Pain intensity (NRS) Disability (ODI)	Anxiety and depression (Hopkins Symptoms Checklist); fear-avoidance (FABQ)	Baseline and 3 and 12 months	Intragroup SSD for both groups in NRS and ODI (*p* < 0.05) Between-group SSD in favor of IG at 3 and 12 months in NRS and ODI (*p* < 0.001)
Fersum et al., 2019 [55]	RCT	IG: *n* = 30 (16 F, 14 M); Age = 42.9 ± 10.9; BMI = 25.6 ± 4.1 CG: *n* = 33 (17 F, 16 M); Age = 43.1 ± 12.8; BMI = 25.1 ± 3.7	Same as Fersum et al., 2013 [54]	Same as Fersum et al., 2013 [54]	Same as Fersum et al., 2013 [54]	Same as Fersum et al., 2013 [54]	Baseline and 3 years	Between-group SSD in favor of the IG at 3 years in ODI (*p* < 0.001)
Gibbs et al., 2018 [50]	RCT	IG: *n* = 13 (11 F, 2 M); Age = 52 ± 9; BMI = 31.0 ± 7.5 CG: *n* = 14 (10 F, 4 M); Age = 51 ± 13; BMI = 29.0 ± 5.2	Intervention on sedentary behavior (face-to-face counseling and by phone + education + use of sit-stand desk + cognitive–behavioral therapy)	No Intervention	Pain intensity (VAS) Disability (ODI)	Physical activity (Global Physical Activity Questionnaire); functionality (functional tests)	Baseline and 3 and 6 months	Intragroup SSD in both groups in ODI at 6 months (*p* < 0.05) Between-group SSD in favor of the IG in ODI at all follow-up moments (*p* < 0.001)
Járomi et al., 2018 [49]	RCT	IG: *n* = 67 (62 F, 5 M); Age = 41.73 ± 3.54; BMI = 24.7 ± 1.84; PD = 25.94 ± 9.36 weeks CG: *n* = 70 (66 F, 4 M); Age = 41.05 ± 3.8; BMI = 24.61 ± 1.78; PD = 27.22 ± 10.60 weeks	Back School education program + general therapeutic exercise	Lifestyle guidance	Pain intensity (VAS)	None	Baseline and 3 months	Intragroup SSD for IG in VAS (*p* < 0.001) Between-group SSD in favor of the IG in VAS (*p* < 0.001)
Kell et al., 2009 [46]	RCT	IG 1: *n* = 9 (3 F, 6 M); Age = 40.1 ± 8.7 IG 2: *n* = 9 (4 F, 5 M); Age = 36.7 ± 8.9 CG: *n* = 9 (4 F, 5 M); Age = 35.3 ± 7.3 Total PD = 27.6 (range 6–96) months	IG 1: Upper and lower limb resistance exercise IG 2: Aerobic exercise	No intervention	Pain intensity (VAS) Disability (ODI)	Quality of life (SF-36); functionality (functional tests)	Baseline and 4 months	Intragroup SSD for IG 1 in VAS and ODI (*p* < 0.05) Between-group SSD in favor of IG 1 vs. IG 2 and CG in ODI and VAS (*p* < 0.05) and IG 2 vs. CG in ODI (*p* < 0.05)
Khodadad et al., 2020 [47]	RCT	IG 1: *n* = 17; Age = 44.3 ± 1.43; BMI = 23.3 ± 1.17 IG 2: *n* = 17; Age = 42.2 ± 3.78; BMI = 24.8 ± 1.65 CG: *n* = 18; Age = 44.4 ± 2.17; BMI = 23.6 ± 1.32	IG 1: Cognitive–functional therapy (education + exercise + mindfulness) IG 2: Lumbar stabilization exercise	Usual physical therapy	Pain intensity (VAS) Disability (ODI)	Behavioral risk factors (obesity, smoking, physical inactivity and risky alcohol consumption)	Baseline and 2 months	Intragroup SSD for IG 1 and IG 2 in VAS (*p* = 0.003) No between-group SSD
Krein et al., 2013 [56]	RCT	IG: *n* = 111 (12 F, 99 M); Age = 51.2 ± 12.5; BMI = 30.6 ± 5.7 CG: *n* = 118 (17 F, 101 M); Age = 51.9 ± 12.8; BMI = 31.6 ± 5.5	Pedometer-based internet-mediated walking program (goal setting + feedback + e-community)	Pedometer-based walking program	Pain intensity (NRS) Disability (RMDQ)	Physical activity (pedometer); fear-avoidance (FABQ); self-efficacy for exercise (Exercise Regularly Scale)	Baseline and 6 and 12 months	Between-group in favor of IG only in RMDQ at 6 months (*p* = 0.02)
Ma et al., 2021 [43]	Non-randomized controlled clinical trial	IG: *n* = 73 (36 F, 37 M); Age = 36.3 ± 6.7; BMI = 23.52 ± 3.05 CG: *n* = 63 (29 F, 34 M); Age = 37.2 ± 7.5; BMI = 24.46 ± 4.72	Maitland training (abdominal stabilization) + pain self-management	Pain self-management	Pain intensity (VAS) Disability (ODI + QLBPRS)	None	Baseline and 3 months	Between-group SSD in favor of the IG in VAS and ODI (*p* < 0.05)
Notarnicola et al., 2013 [45]	Prospective observational study	Total: *n* = 60 (33 F, 27 M); Age = 51.2 ± 9.8	Pilates exercise program	No intervention	Disability (RMDQ + ODI)	Quality of life (SF-36); Ability to perform work tasks (Spinal Functional Sort)	Baseline and 6 months	Intragroup SSD for IG in RMDQ and ODI (*p* < 0.001) Between-group SSD in favor of IG in ODI (*p* = 0.006)
Phattharas-upharerk et al., 2019 [51]	RCT	IG: *n* = 36 (24 F, 12 M); Age = 35.7 ± 3.6 CG: *n* = 36 (22 F, 14 M); Age = 34.8 ± 4.3	Qigong	Waiting list (general advice on pain self-management)	Pain intensity (VAS) Disability (RMDQ)	Mental status (Srithanya Stress Scale)	Baseline and 6 weeks (follow-up every week)	Intragroup SSD for IG in VAS (*p* < 0.001) Between-group SSD in favor of the IG in VAS and RMDQ (*p* < 0.001 and *p* = 0.022, respectively)
Sherman et al., 2011 [59]	RCT	IG 1: *n* = 92 (62 F, 30 M); Age = 46.6 ± 9.8 IG 2: *n* = 91 (57 F, 34 M); Age = 49 ± 9.91 CG: *n* = 45 (27 F, 18 M); Age = 50.8 ± 9.07	IG 1: Yoga IG 2: General exercise	The Back Pain Helpbook	Pain intensity (NRS) Disability (RMDQ)	None	Baseline, 3 months and 26 weeks	Intragroup SSD for all groups in RMDQ at all follow-up times and in VAS at 12 weeks Between-group SSD in favor of IG 1 vs. CG in RMDQ and in VAS; between-group SSD in favor of IG 2 vs. CG only in RMDQ; without DES between both IG
Sofi et al., 2011 [42]	Non-randomized community trial	Total: *n* = 650 (560 F, 90 M); Age: 65 (range 23–87); BMI: 26.5 ± 4.2	Empoli Adaptive Physical Activity	-	Pain intensity (VAS)	Functionality (Short Physical Performance Battery)	Baseline and 12-months	Between-group SSD between adherence and non-adherence for the VAS (*p* < 0.0001)
Tekur et al., 2008 [57]	RCT	IG: *n* = 40 (21 F, 19 M); Age = 49 ± 3.6 CG: *n* = 40 (15 F, 25 M); Age = 48 ± 4	Yoga + pain self-management	Mobility exercise + pain self-management	Disability (ODI)	None	Baseline and 1 week	Intragroup SSD for IG in ODI (*p* = 0.001) Between-group SSD in favor of the GI in ODI (*p* < 0.001)
Tekur et al., 2012 [58]	RCT	Same as Tekur et al., 2008 [57]	Same as Tekur et al., 2008 [57]	Same as Tekur et al., 2008 [57]	Pain intensity (NRS)	Anxiety (State-Trait Anxiety Inventory); depression (Beck’s depression inventory); functionality (sit and reach)	Baseline and 1 week	Intragroup SSD for both groups in VAS (IG: *p* < 0.001 and CG: *p* = 0.005) Between-group SSD in favor of the IG in VAS (*p* < 0.001)
Williams et al., 2018 [52]	RCT	IG: *n* = 79 (48 F, 31 M); Age = 56.0 ± 13.3; BMI = 32.4 ± 3.5 CG: *n* = 80 (46 F, 34 M); Age = 57.4 ± 13.6; BMI = 32.1 ± 3.6	Healthy lifestyle promotion (brief telephone advice + offer of a clinical consultation + referral to a telephone-based health coaching service)	Waiting list	Pain intensity (NRS) Disability (RMDQ)	Quality of life (SF-12); sleep quality (PSQI); physical activity (Active Australia Survey); depression and anxiety (DASS); fear-avoidance (FABQ); behavioral risk factors	Baseline and 6 months (follow-up every month)	Between-group SSD in favor of IG in NRS only at 10 and 18 weeks (*p* = 0.05 and *p* = 0.01, respectively)
Zou et al., 2019 [60]	RCT	IG 1: *n* = 15 (11 F, 4 M); Age = 58.13 ± 5.38 IG 2: *n* = 15 (11 F, 4 M); Age = 58.4 ± 5.08 CG: *n* = 13 (10 F, 3 M); Age = 60.67 ± 2.58	IG 1: Tai Chi IG 2: Core stability training	No intervention	Pain intensity (VAS)	None	Baseline and 3 months	Between-group SSD in favor of IG 1 (*p* < 0.01) vs. CG and IG 2 vs. CG (*p* < 0.01)

ADLs, Activities of Daily Living; Age, mean age in years; BMI, Body Mass Index (kg/m^2^); CG, control group; DASS, Depression, Anxiety and Stress Scale; F, female; FABQ, Fear-Avoidance Beliefs Questionnaire; IG, intervention group; IPAQ, International Physical Activity Questionnaire; M, male; NRS, Numeric Rating Scale 11-point; ODI, Oswestry Disability Index; PD, pain duration (mean in weeks); PSQI, Pittsburgh Sleep Quality Index; QLBPRS, Quebec Low Back Pain Rating Scale; RCT, randomized controlled trial; RMDQ, Roland–Morris Disability Questionnaire; SF-12, Short-Form 12-Item Health Survey; SF-36: Short-Form 36-Item Health Survey; SSD, significant statistical difference (*p*-value < 0.05); VAS, Visual Analogue Scale. Data are presented as mean ± standard deviation.

**Table 2:** Assessment of methodological quality by PEDro scale.

Author and Year	1	2	3	4	5	6	7	8	9	10	11	Total	Quality
Amorim et al., 2019 [53]	YES	YES	YES	YES	NO	NO	YES	NO	YES	YES	YES	7	High
Bendix et al., 1998 [31]	YES	YES	NO	YES	NO	NO	NO	YES	NO	YES	YES	5	Moderate
Cuesta-Vargas et al., 2011 [48]	YES	YES	NO	YES	NO	YES	YES	YES	NO	YES	YES	7	High
Fersum et al., 2013 [54]	YES	YES	YES	YES	NO	NO	YES	NO	YES	YES	YES	7	High
Fersum et al., 2019 [55]	YES	YES	YES	YES	NO	NO	YES	NO	YES	YES	YES	7	High
Gibbs et al., 2018 [50]	YES	YES	NO	YES	NO	NO	YES	YES	NO	YES	YES	6	High
Járomi et al., 2018 [49]	YES	YES	NO	YES	NO	NO	YES	YES	NO	YES	YES	6	High
Kell et al., 2009 [46]	YES	YES	NO	YES	NO	NO	NO	NO	NO	YES	YES	4	Moderate
Khodadad et al., 2020 [47]	YES	YES	YES	YES	NO	NO	YES	YES	NO	YES	YES	7	High
Krein et al., 2013 [56]	YES	YES	YES	YES	NO	NO	NO	YES	YES	YES	YES	7	High
Phattharasupharerk et al., 2019 [51]	YES	YES	YES	YES	NO	NO	YES	YES	YES	YES	YES	8	High
Sherman et al., 2011 [59]	YES	YES	YES	YES	NO	NO	NO	YES	YES	YES	YES	7	High
Tekur et al., 2008 [57]	YES	YES	YES	YES	NO	NO	YES	YES	NO	YES	YES	7	High
Tekur et al., 2012 [58]	YES	YES	YES	YES	NO	NO	NO	YES	NO	YES	YES	6	High
Williams et al., 2018 [52]	YES	YES	YES	YES	NO	NO	YES	YES	YES	YES	YES	8	High
Zou et al., 2019 [60]	YES	YES	NO	YES	NO	NO	YES	YES	NO	YES	YES	6	High

NO, the study does not present the studied criterion; YES, the study presents the studied criterion; 1, eligibility criteria were specified (this factor does not contribute the final score); 2, subjects were randomly assigned to groups; 3, allocation was concealed; 4, the groups had similar baseline characteristics regarding the most important prognostic indicators; 5, blinding was implemented for all subjects; 6, blinding was implemented for all therapists administering the therapy; 7, blinding was implemented for all assessors measuring at least one key outcome; 8, measures for at least one key outcome were obtained from over 85% of subjects initially allocated to groups; 9, all subjects with available outcome measures received the treatment or control condition as allocated, or if not, data for at least one key outcome were analyzed using “intention to treat”; 10, between-group statistical comparisons for at least one key outcome are reported; 11, the study offers both point measures and measures of variability for at least one key outcome.

Of the three studies evaluated with the ROBINS-I tool, two were found to have an overall serious risk [42,44], while the other study did not have enough information about deviations from intended interventions, missing data and measurement of outcomes [43] (Table 3).

Lastly, the observational study [45] evaluated with the NHLBI tool was found to be of fair quality (Table 4).

### 3.4. Review Results

#### 3.4.1. Pain Intensity

Multimodal intervention programs that integrated at least therapeutic exercise, pain self-management and patient education were those that obtained the best results between groups to decrease pain intensity [31,43,58]. Within the multimodal programs, it is worth highlighting the effectiveness of cognitive–functional therapy in reducing the pain intensity, which addresses the different aspects of the painful experience (physical, emotional and social) [47,54].

Exercise programs, regardless of their modality, were also effective, mainly when compared with not performing any intervention [44,46,47,51,59,60].

Lastly, other interventions that also showed improvements in pain intensity were programs aimed at promoting physical activity, healthy lifestyle (focused on lifestyle risks such as overweight, nutrition, smoking, alcohol and poor sleep quality) and reduced sedentary time [42,52,53].

#### 3.4.2. Functional Disability

The results obtained in terms of functional disability were like the ones described for pain intensity, although with some differences in the study by Fersum et al. [55], where it was observed that the improvements produced in the cognitive–functional therapy group were maintained for up to 3 years of follow-up.

### 3.5. Meta-Analysis Results

In the multivariate NMA, the ANOVA test was significant (LRT = 58.16, *p* < 0.001), which indicates that the fit of the complete random-effects model is better than the reduced fixed-effects model, also presenting lower values in both the BIC (443.30 vs. 453.50) and AIC (430.17 vs. 448.33). In the univariate NMAs under a fixed effects model, the inconsistency between studies was significant, both in the pain intensity outcome (X^2^(5) = 373.32, *p* < 0.001) and functional disability (X^2^(1) = 50.32, *p* < 0.001). When consistency was assessed under the assumption of a complete random-effects model of interaction design per treatment, the inconsistency between studies remained significant, although the value of the statistic decreased its value in the pain intensity outcome (X^2^(5) = 8.31, *p* = 0.14); meanwhile, in functional disability, it decreased and become non-significant (X^2^(1) = 31.79, *p* < 0.001), indicating that the random-effects model is the most adequate to adjust, at least in part, for the inconsistency and heterogeneity of the analysis.

The network graphs with the size of the interventions weighted by the covariates show how the age distribution is similar throughout the comparisons, whereas in the case of the male/female ratio, when comparisons include cognitive therapy (including any treatment focused on some cognitive component, such as behavior or lifestyle change, motivation, positive feedback, etc.) or health information (limited to advice and recommendations), it cannot be ensured that the effects in these comparisons are not influenced by this confounding variable (Supplementary Figure S1).

In the multivariate NMA, the significant Cochrane Q test (X^2^(11) = 438.21, *p* < 0.001) evidenced the presence of heterogeneity. In pain intensity, significant heterogeneity was observed both in the VAS, with a value of I^2^ = 93.36% (τ^2^ = 0.083), and in NRS, with a value of I^2^ = 93.52% (τ^2^ = 0.085), as in functional disability both in the ODI, with a value of I^2^ = 93.45% (τ^2^ = 0.084), and in the RMQD, with a value of I^2^ = 93.40% (τ^2^ = 0.083). In the univariate NMAs, in the pain intensity outcome, the Cochrane Q test was significant (X^2^(8) = 1728.15, *p* < 0.001), with a value of I^2^ = 99.54%, 95%CI (99.44%, 99.618%) (τ^2^ = 199.50), indicating significant global heterogeneity. On the other hand, intra-study heterogeneity (X^2^(3) = 1354.82, *p* < 0.001) was also significant. In the functional disability outcome, the Cochrane Q test was significant (X^2^(3) = 53.94, *p* < 0.001), with a value of I^2^ = 94.44%, 95%CI (88.86%, 97.22%) (τ^2^ = 34.20), indicating large and significant global heterogeneity. On the other hand, intra-study heterogeneity (X^2^(2) = 3.62, *p* = 0.164) was not significant.

In the univariate NMAs, the net-splitting method did not show significant differences (*p* > 0.05) between the direct and indirect estimates both in the network for pain intensity and for functional disability, which seems to point to sufficient consistency in the comparisons made (Supplementary Table S2). The graphs of direct and indirect comparisons show a high percentage of indirect comparisons in the total estimate of each comparison, both in the outcome pain intensity and in functional disability. It was the indirect comparisons that presented a mean path length greater than 2, indicating that they may be the cause of poorer compliance with the assumptions of the model (Supplementary Figure S2). In the multivariate NMA, it was evident how the studies of Sherman et al. [59] and Kell et al. [46] are the ones that contribute the most to heterogeneity (Supplementary Figure S3). In the univariate NMAs, no direct comparison influenced more than 1% of the total mixed comparisons for both pain intensity and functional disability, so it is not likely that the methodological quality of the individual articles is biasing the consistency of the analysis (Supplementary Table S3).

**Figure 3:**
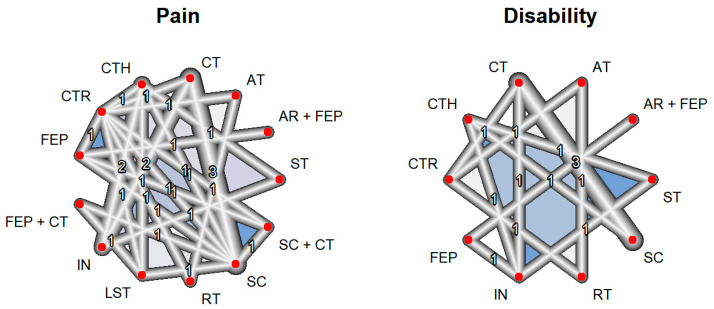
Network constructed for outcomes. AR, aquatic running; AT, aerobic training; CT, cognitive therapy; CTH, complementary therapy; CTR, control; FEP, functional exercise program; IN, health information; LST: lumbar stabilization training; RT, resistance training; SC, standard care; ST, stretching. Each of the numbers represents how many comparisons have been carried out between the corresponding interventions in the included studies.

Pain intensity was measured in 14 studies with a total of 13 interventions and 27 pairs of comparisons, including a total of 1466 patients. On the other hand, the functional disability outcome included 10 studies with a total of 10 interventions and 14 pairs of comparisons, including a total of 1044 patients (Table S4). In both the pain intensity and functional disability outcomes, the network graph shows that the largest number of studies compared standard care (manual therapy plus general exercise) vs. cognitive therapy (Figure 3).

**Figure 4:**
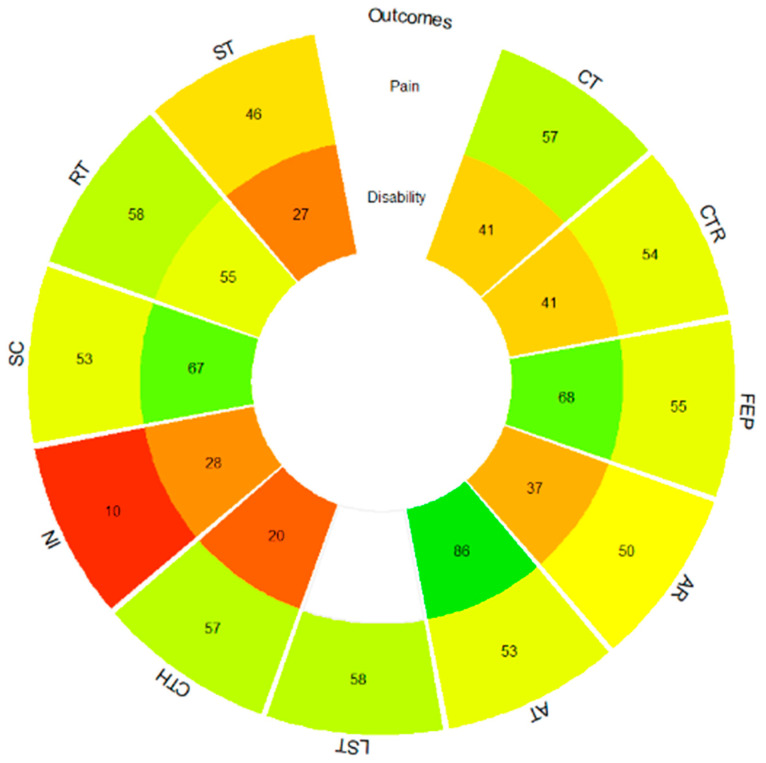
Pain and functional disability P-score rank-heat plot. AR, aquatic running; AT, aerobic training; CT, cognitive therapy; CTH, complementary therapy; CTR, control; FEP, functional exercise program; IN, health information; LST, lumbar stabilization training; RT, resistance training; SC, standard care; ST, stretching. Colors: higher green, higher P-Score, higher red, lower P-score.

The ranking of treatments for multivariate NMA shows how functional exercise programs (most of them include a multimodal approach, together with pain self-management strategies and patient education, mainly based on neuroscience of pain), followed by the combination of aquatic exercise with functional exercise programs and aerobic exercise, present the highest P-score values and, therefore, improve both pain intensity and functional disability in a combined way (Supplementary Table S5). In the univariate NMAs, the ranking of pain treatments shows how cognitive therapy, followed by lumbar stabilization exercises and resistance training, presents the highest P-score values and can be considered the most effective in reducing pain intensity. In the case of functional disability, it is the functional exercise programs, followed by aerobic training and standard care, which present the highest values in the P-score and can be considered the most effective in reducing the level of functional disability (Figure 4 and Supplementary Table S5). The rankogram with the SUCRA values shows how lumbar stabilization exercises, followed by the combination of functional exercise programs with cognitive therapy and resistance training, are the ones that contribute the most to pain intensity reduction, while functional exercise programs, followed by aerobic training and standard care, are those that reduce functional disability in the greatest proportion (Supplementary Figure S4).

On the one hand, in the multivariate NMA, significant differences between treatments were evident in several comparisons for pain intensity and functional disability (Supplementary Table S6). On the other hand, in the univariate NMAs, in the pain intensity outcome, the league table does not show significant differences except between cognitive therapy vs. health information and complementary therapy vs. health information with higher reductions in the former against health information (Table 5). For the functional disability outcome, again, several significant comparisons were found (Table 5).

On the pain intensity outcome, the Egger (t(25) = −0.19, *p* = 0.854) and Thompson–Sharp tests (t(25) = −1.77, *p* = 0.088) were not significant, while the Begg–Mazumdar test (Z = 3.19, *p* = 0.001) was significant. In the funnel plot, only the comparisons cognitive therapy vs. health information and complementary therapy vs. health information were outside the significance bands, so it is concluded that there is no publication bias. In the case of functional disability, the Egger (t(12) = 0.88, *p* = 0.397), Begg–Mazumdar (Z = −1.92, *p* = 0.055) and Thompson–Sharp tests (t(12) = −1.77, *p* = 0.39) were not significant. In the funnel plot, only the comparison cognitive therapy vs. standard care was outside the significance bands, so there appears to be no publication bias (Supplementary Figure S5).

## 4. Discussion

The aim of this systematic review and meta-analysis was to examine which lifestyle interventions could lead to better improvements in pain intensity and functional disability in people suffering from CNSLBP. For this purpose, 20 studies of moderate-to-high methodological quality and heterogeneous risk of bias were included in the systematic review for qualitative analysis, of which 16 were RCTs and went on to conduct a more exhaustive quantitative analysis through the univariate and multivariate network meta-analysis combining pain intensity and functional disability outcomes.

Both the results of the systematic review and of the different statistical techniques of the meta-analysis seem to point in the same direction. Firstly, the intervention that showed the best results to improve pain intensity in the population with CNSLBP has turned out to be cognitive therapy combined with functional exercise programs, that is, interventions aimed at changing lifestyle and behavior that include therapeutic exercise, pain education and the promotion of patient self-management strategies, followed by lumbar stabilization exercise and resistance exercise. Secondly, to improve functional disability, functional exercise programs seem to be the most effective again, followed by aerobic exercise and standard care (manual therapy plus general exercise). Thirdly, regarding the improvement of both outcomes jointly, which is usually one of the most frequent objectives to achieve in clinical practice with patients with CNSLBP [64,65], functional exercise programs, followed by aquatic and aerobic exercise, were the interventions that had achieved the best results.

There is a wide variety of NMAs that have analyzed the effectiveness of different interventions in the LBP population. The vast majority of these has focused on which exercise modalities are the most effective in improving pain intensity and functional disability, also analyzing the influence of different psychological interventions. Within the exercise modalities, based on the results obtained from our study, lumbar and core stabilization exercises, resistance exercises, aerobic exercise, stretching, qigong, yoga, Pilates and tai chi are recommended; highlighted among these are resistance exercises, aerobic exercises and stabilization/control motor exercises due to the greater benefits shown. In the aforementioned NMAs, we can find heterogeneous results, although most of them agree with our results. On the one hand, in several NMAs, resistance and stabilization exercises are the two modalities that have proven to be amongst the most effective [66,67,68] with Pilates [66,67], which is considered one of the most effective mind–body exercise modalities, followed by tai chi, yoga and qigong [69]. On the other hand, aerobic exercise has only been recommended as one of the most effective exercise modalities in the NMA by Owen et al. [66]. This discrepancy in the results, as well as the fact that stretching and standard care, both of which consist to an extent of physical exercise and movement, were among the most effective interventions in our study, is consistent with the current scientific literature, which recommends exercise as the first line of management for LBP, but it has not yet shown that a single exercise modality by itself is considered superior to another [70].

In relation to the NMAs that also included psychological interventions, aimed at reducing pain related distress and functional disability by changing patients’ negative beliefs, behaviors and attitudes through a combination of principles and strategies informed by psychological theories, pain education and cognitive–behavioral therapy stand out as the most effective treatments, but they all agree that these interventions are more effective when carried out in conjunction with physical therapy programs (mainly structured therapeutic exercise) [71,72,73]. These results are in line with the intervention that produced the best effect for both pain intensity and functional disability in our study, a multimodal intervention that addresses cognitive, behavioral and physical aspects. Chronic pain conditions such as CNSLBP require multimodal treatment approaches that address biopsychosocial dimensions [74], as they are imperative to identify the altered cognitions, maladaptive beliefs and behaviors that are contributing to each patient’s pain and functional disability before commencing exercise [75] Even though education, self-management strategies and the incorporation of elements of important lifestyle behaviors (i.e., physical activity participation, sleep quality and mood) are considered first-line care for CNSLBP [12], physical therapists are still not fully aware and do not fully integrate support regarding important self-management skills, considering the patient’s expectations and psychosocial factors as the most important barriers [76].

Regarding interventions that solely focused on promoting physical activity, lifestyle changes and/or reducing sedentary time, it was found that the variables which were shown to improve pain intensity and functional disability outcomes were motivation, positive feedback and a follow-up by the professional throughout the recovery process and functional improvement. In general, simply providing health information to the patient seems insufficient and has proven to be one of the interventions with the worst results for improving pain intensity and functional disability in our study. This may be partly because people with CNSLBP have reported greater difficulty in engaging in general positive health behaviors [77]; thus, without external help from a health professional, this change will be difficult to implement.

The main limitations of this NMA are, on the one hand, the influence that the male/female ratio may have and, on the other hand, the significant heterogeneity found, probably due to the variability between studies (characteristics of the participants, type and duration of the intervention, measurement tools, etc.), as well as the presence of a high risk of bias in a significant proportion of the included RCTs. This study also has strengths, such as having performed the NMA with 14 studies and more than 1400 people for the pain intensity outcome and 10 studies and more than 1000 people for functional disability, together with the fact that there is no publication bias among all of these studies. Moreover, all the statistically significant differences found and discussed throughout the study provide validity and value to it.

As far as we are aware, this is the first initial systematic review and meta-analysis aimed at assessing the effectiveness of lifestyle-related interventions in addressing pain intensity and functional disability among individuals with CNSLBP. For future research, given that this NMA has simply evaluated the effectiveness before and after treatment, that is, in the short term, it would be interesting to be able to evaluate the effects that different interventions have in the long term in terms of pain intensity, relapses and exacerbations, self-management capacity and functionality in the person’s daily life activities. In relation to the influence of sex on the results, performing some type of analysis by subgroups could be considered, as men and women have shown to have different responses to treatment and perceptions of pain [78], or even consider including in future clinical practice guidelines on the management of CNSLBP aspects to be considered depending on the sex of the person suffering from this condition.

## 5. Conclusions

A multimodal intervention aimed at changing lifestyle-encompassing cognitive, behavioral and physical aspects appears to be the most effective in improving pain intensity and functional disability caused by CNSLBP; however, it has not yet been demonstrated that one exercise modality alone can emphasize all of these aspects or that one type of exercise is superior to another. Moreover, it is not yet known whether these improvements are sustained over the long term. On the other hand, feedback, motivation and follow-up appear to be key factors in these interventions. However, future research is needed, especially well-designed longitudinal studies to better elucidate the long-term efficacy of multimodal interventions.

## Figures and Tables

**Table 3:** Assessment of risk of bias in studies with non-randomized intervention by ROBINS-I.

Author and Year	D1	D2	D3	D4	D5	D6	D7	Overall Risk of Bias
Baena-Beato et al., 2013 [44]	Serious	Low	Low	Low	Moderate	Low	Low	Serious
Ma et al., 2021 [43]	Serious	Low	Serious	No information	No information	No information	Moderate	No information
Sofi et al., 2011 [42]	Moderate	Low	Low	Moderate	Moderate	Serious	Moderate	Serious

D1, confounding-induced bias; D2, participant selection bias; D3, intervention classification bias; D4, bias from deviations in intended interventions; D5, missing-data bias; D6, outcome measurement bias; D7, reported result selection bias. Overall risk of bias: low (low risk across all domains), moderate (low or moderate risk across all domains), serious (serious risk in at least one domain, but not at critical level in any domain), critical (critical risk in at least one domain) and no information (unclear indication of serious or critical bias, with a lack of information in one or more key domains of bias).

**Table 4:** Assessment of methodological by NHLBI Quality Assessment Tool for Observational Cohort and Cross-Sectional Studies.

Author and Year	Q1	Q2	Q3	Q4	Q5	Q6	Q7	Q8	Q9	Q10	Q11	Q12	Q13	Q14	Quality Rating
Notarnicola et al., 2013 [45]	YES	YES	NR	YES	NO	YES	YES	NO	YES	NO	YES	YES	YES	YES	Fair

CD, cannot determine; NA, not applicable; NO, the answer to the corresponding question is no; NR, not reported; YES, the answer to the corresponding question is yes. Q1: Was the research question or objective in this paper clearly stated? Q2: Was the study population clearly specified and defined? Q3: Was the participation rate of eligible persons at least 50%? Q4: Were all the subjects selected or recruited from the same or similar populations (including the same time period)? Were inclusion and exclusion criteria for being in the study prespecified and applied uniformly to all participants? Q5: Was a sample size justification, power description, or variance and effect estimates provided? Q6: For the analyses in this paper, were the exposure(s) of interest measured prior to the outcome(s) being measured? Q7: Was the timeframe sufficient so that one could reasonably expect to see an association between exposure and outcome if it existed? Q8: For exposures that can vary in amount or level, did the study examine different levels of the exposure as related to the outcome (e.g., categories of exposure, or exposure measured as continuous variable)? Q9: Were the exposure measures (independent variables) clearly defined, valid, reliable and implemented consistently across all study participants? Q10: Was the exposure(s) assessed more than once over time? Q11: Were the outcome measures (dependent variables) clearly defined, valid, reliable and implemented consistently across all study participants? Q12: Were the outcome assessors blinded to the exposure status of participants? Q13: Was loss to follow-up after baseline 20% or less? Q14: Were key potential confounding variables measured and adjusted statistically for their impact on the relationship between exposure(s) and outcome(s)?

**Table 5:** League table reporting the comparative effects for all interventions for the pain intensity network.

Pain Intensity Outcome												
**CT**												
−1.38 (−19.34, 16.59)	**CTR**											
−0.88 (−28.36, 26.60)	0.49 (−25.01, 25.99)	**FEP**										
−2.78 (−42.83, 37.27)	−1.41 (−40.13, 37.31)	−1.90 (−31.04, 27.24)	**AR + FEP**									
−1.18 (−34.182, 31.83)	0.20 (−27.49, 27.89)	−0.29 (−37.93, 37.35)	1.61 (−45.99, 49.21)	**AT**								
0.12 (−28.41, 28.65)	1.50 (−27.78, 30.77)	1.00 (−34.58, 36.59)	2.90 (−43.09, 48.89)	1.30 (−39.00, 41.59)	**FEP + CT**							
0.16 (−21.94, 22.27)	1.54 (−19.95, 23.03)	1.05 (−29.44, 31.53)	2.95 (−39.22, 45.11)	1.34 (−33.71, 36.39)	0.04 (−25.92, 26.00)	**LST**						
−0.57 (−18.58, 17.45)	0.81 (−18.79, 20.41)	0.31 (−28.72, 29.35)	2.21 (−38.92, 43.35)	0.61 (−33.32, 34.53)	−0.69 (−29.97, 28.60)	−0.73 (−22.23, 20.77)	**CTH**					
−19.42 (−35.96, −2.87) ^a^	−18.04 (−39.64, 3.56)	−18.53 (−48.73, 11.67)	−16.63 (−58.60, 25.33)	−18.24 (−53.36, 16.88)	−19.54 (−50.34, 11.27)	−19.58 (−43.85, 4.69)	−18.85 (−35.52, −2.18) ^a^	**IN**				
−1.72 (−15.37, 11.92)	−0.35 (−17.65, 16.95)	−0.84 (−26.34, 24.66)	1.06 (−37.66, 39.78)	−0.55 (−33.20, 32.10)	−1.84 (−27.80, 24.12)	−1.89 (−21.76, 17.99)	−1.15 (−18.48, 16.17)	17.69 (−1.17, 36.56)	**SC**			
−1.65 (−29.14, 25.83)	−0.28 (−25.78, 25.22)	−0.77 (−28.47, 26.93)	1.13 (−39.07, 41.33)	−0.48 (−38.12, 37.17)	−1.77 (−37.36, 33.81)	−1.82 (−32.30, 28.67)	−1.08 (−30.12, 27.95)	17.76 (−12.44, 47.97)	0.07 (−25.44, 25.58)	**SC + CT**		
0.62 (−32.38, 33.63)	2.00 (−25.69, 29.69)	1.51 (−36.13, 39.15)	3.41 (−44.19, 51.01)	1.80 (−25.89, 29.49)	0.51 (−39.79, 40.80)	0.46 (−34.59, 35.51)	1.19 (−32.73, 35.12)	20.04 (−15.08, 55.16)	2.35 (−30.30, 35.00)	2.28 (−35.37, 39.92)	**RT**	
−10.15 (−38.52, 18.21)	−8.78 (−39.29, 21.73)	−9.27 (−46.45, 27.92)	−7.37 (−54.61, 39.87)	−8.98 (−50.18, 32.23)	−10.27 (−47.80, 27.26)	−10.31 (−42.43, 21.80)	−9.58 (−34.97, 15.80)	9.27 (−16.12, 34.65)	−8.43 (−37.30, 20.44)	−8.50 (−45.69, 28.69)	−10.78 (−51.98, 30.43)	**ST**
**Functional disability outcome**												
**CT**												
−1.52 (−22.28, 19.24)	**AR + FEP**											
16.40 (0.00, 32.80) ^a^	17.92 (−8.53, 44.37)	**AT**										
0.30 (−11.25, 11.85)	1.82 (−21.93, 25.57)	−16.10 (−27.74, −4.46) ^a^	**CTR**									
32.88 (17.05, 48.72) ^a^	34.40 (20.98, 47.82) ^a^	16.48 (−6.32, 39.28)	32.58 (12.98, 52.18) ^a^	**FEP**								
−5.14 (−15.83, 5.55)	−3.62 (−24.36, 17.13)	−21.54 (−41.11, −1.96) ^a^	−5.44 (−21.17, 10.30)	−38.02 (−53.84, −22.20) ^a^	**CTH**							
−3.22 (−13.00, 6.56)	−1.70 (−20.01, 16.61)	−19.62 (−38.71, −0.53) ^a^	−3.52 (−18.65, 11.61)	−36.10 (−48.56, −23.64) ^a^	1.92 (−7.84, 11.67)	**IN**						
5.69 (−0.91, 12.30)	7.21 (−13.92, 28.34)	−10.71 (−28.40, 6.97)	5.39 (−7.91, 18.69)	−27.19 (−43.51, −10.87) ^a^	10.83 (0.72, 20.94) ^a^	8.91 (−1.64, 19.46)	**SC**					
4.10 (−12.30, 20.50)	5.62 (−20.84, 32.08)	−12.30 (−23.88, −0.72) ^a^	3.80 (−7.85, 15.45)	−28.78 (−51.58, −5.98) ^a^	9.24 (−10.34, 28.82)	7.32 (−11.78, 26.42)	−1.59 (−19.28, 16.09)	**RT**				
−3.84 (−17.28, 9.59)	−2.32 (−23.74, 19.09)	−20.24 (−41.45, 0.96)	−4.14 (−21.86, 13.57)	−36.72 (−53.41, −20.03) ^a^	1.29 (−9.80, 12.38)	−0.62 (−11.73, 10.48)	−9.54 (−23.04, 3.97)	−7.94 (−29.15, 13.26)	**ST**			

AR, aquatic running; AT, aerobic training; CT, cognitive therapy; CTH, complementary therapy; CTR, control; FEP, functional exercise program; IN, health information; LST, lumbar stabilization training; RT, resistance training; SC, standard care; ST, stretching. Significant differences are represented by ^a^.

## Data Availability

The datasets generated during and/or analyzed during the current study are available from the corresponding author upon reasonable request.

## Supplementary Materials

The following supporting information can be downloaded at https://www.mdpi.com/article/10.3390/healthcare12050505/s1, Figure S1: Covariate-weighted networks; Figure S2: Direct evidence proportion for each network estimate (random-effects model); Figure S3: Heterogeneity studies contribution plot; Figure S4: Pain intensity and functional disability SUCRA rankogram plots; Figure S5: Publication bias funnel plots; Table S1: PRISMA Checklist; Table S2: Direct–indirect estimates; Table S3: Contribution studies table; Table S4: Characteristics of studies; Table S5: Ranking table of interventions; Table S6: Pairwise comparison for combined pain intensity–functional disability.
