# Supplementary material for: Effects of Lifestyle Interventions on the Improvement of Chronic Non-Specific Low Back Pain: A Systematic Review and Network Meta-Analysis
Source: Healthcare (Basel). 2024 Feb 20;12(5):505. doi: 10.3390/healthcare12050505 (PMC10931043; doi:10.3390/healthcare12050505)
Supplement: Supplementary file 1 [file healthcare-12-00505-s001.zip › healthcare-2821410-supplementary.pdf]

SUPPLEMENTARY MATERIAL

Table S1. PRISMA Checklist.

Section and Topic	Item #	Checklist item	Location where item is reported
**TITLE**			
Title	1	Identify the report as a systematic review.	1
**ABSTRACT**			
Abstract	2	See the PRISMA 2020 for Abstracts checklist.	1
**INTRODUCTION**			
Rationale	3	Describe the rationale for the review in the context of existing knowledge.	1-2
Objectives	4	Provide an explicit statement of the objective(s) or question(s) the review addresses.	2
**METHODS**			
Eligibility criteria	5	Specify the inclusion and exclusion criteria for the review and how studies were grouped for the syntheses.	3
Information sources	6	Specify all databases, registers, websites, organizations, reference lists and other sources searched or consulted to identify studies. Specify the date when each source was last searched or consulted.	3
Search strategy	7	Present the full search strategies for all databases, registers and websites, including any filters and limits used.	3
Selection process	8	Specify the methods used to decide whether a study met the inclusion criteria of the review, including how many reviewers screened each record and each report retrieved, whether they worked independently and, if applicable, details of automation tools used in the process.	3
Data collection process	9	Specify the methods used to collect data from reports, including how many reviewers collected data from each report, whether they worked independently, any processes for obtaining or confirming data from study investigators and, if applicable, details of automation tools used in the process.	3
Data items	10a	List and define all outcomes for which data were sought. Specify whether all results that were compatible with each outcome domain in each study were sought (e.g., for all measures, time points and analyses), and if not, specify the methods used to decide which results to collect.	3
	10b	List and define all other variables for which data were sought (e.g., participant and intervention characteristics, and funding sources). Describe any assumptions made about any missing or unclear information.	3
Study risk of bias assessment	11	Specify the methods used to assess risk of bias in the included studies, including details of the tool(s) used, how many reviewers assessed each study and whether they worked independently and, if applicable, details of automation tools used in the process.	3-4
Effect measures	12	Specify for each outcome the effect measure(s) (e.g., risk ratio and mean difference) used in the synthesis or presentation of results.	4-5

Synthesis methods	13a	Describe the processes used to decide which studies were eligible for each synthesis (e.g., tabulating the study intervention characteristics and comparing against the planned groups for each synthesis (item #5)).	4-5
	13b	Describe any methods required to prepare the data for presentation or synthesis, such as handling of missing summary statistics, or data conversions.	4
	13c	Describe any methods used to tabulate or visually display the results of individual studies and syntheses.	4
	13d	Describe any methods used to synthesize results and provide a rationale for the choice(s). If a meta-analysis was performed, describe the model(s), method(s) to identify the presence and extent of statistical heterogeneity, and software package(s) used.	4-5
	13e	Describe any methods used to explore possible causes of heterogeneity among study results (e.g., subgroup analysis and meta-regression).	5
	13f	Describe any sensitivity analyses conducted to assess robustness of the synthesized results.	5
Reporting bias assessment	14	Describe any methods used to assess risk of bias due to missing results in a synthesis (arising from reporting biases).	5
Certainty assessment	15	Describe any methods used to assess certainty (or confidence) in the body of evidence for an outcome.	X
**RESULTS**			
Study selection	16a	Describe the results of the search and selection process, from the number of records identified in the search to the number of studies included in the review, ideally using a flow diagram.	5<br>Figure 1
	16b	Cite studies that might appear to meet the inclusion criteria but which were excluded and explain why they were excluded.	Figure 1
Study characteristics	17	Cite each included study and present its characteristics.	6-7<br>Table 1
Risk of bias in studies	18	Present assessments of risk of bias for each included study.	14-16<br>Figure 2<br>Tables 2–4
Results of individual studies	19	For all outcomes, present, for each study, (a) summary statistics for each group (where appropriate) and (b) an effect estimate and its precision (e.g., confidence/credible interval), ideally using structured tables or plots.	Table 1<br>Table S4
Results of syntheses	20a	For each synthesis, briefly summarize the characteristics and risk of bias among contributing studies.	16-17<br>Figure S3,<br>Table S3
	20b	Present the results of all statistical syntheses conducted. If a meta-analysis was performed, present for each the summary estimate and its precision (e.g., confidence/credible interval) and measures of statistical heterogeneity. If comparing groups, describe the direction of the effect.	16-21<br>Figure 4<br>Table 5<br>Figures S2

			and S4, Tables S2 and S4–S6
	20c	Present the results of all investigations of possible causes of heterogeneity among study results.	17<br>Figures S1 and S3, Table S3
	20d	Present the results of all sensitivity analyses conducted to assess the robustness of the synthesized results.	16-21
Reporting biases	21	Present assessments of risk of bias due to missing results (arising from reporting biases) for each synthesis assessed.	21<br>Figure S5
Certainty of evidence	22	Present assessments of certainty (or confidence) in the body of evidence for each outcome assessed.	X
**DISCUSSION**			
Discussion	23a	Provide a general interpretation of the results in the context of other evidence.	21-23
	23b	Discuss any limitations of the evidence included in the review.	22
	23c	Discuss any limitations of the review processes used.	22
	23d	Discuss implications of the results for practice, policy and future research.	22-23
**OTHER INFORMATION**			
Registration and protocol	24a	Provide registration information for the review, including register name and registration number, or state that the review was not registered.	2
	24b	Indicate where the review protocol can be accessed, or state that a protocol was not prepared.	2
	24c	Describe and explain any amendments to information provided at the time of registration or in the protocol.	2
Support	25	Describe sources of financial or non-financial support for the review and the role of the funders or sponsors in the review.	23
Competing interests	26	Declare any competing interests of review authors.	23
Availability of data, code and other materials	27	Report which of the following are publicly available and where they can be found: template data collection forms, data extracted from included studies, data used for all analyses, analytic code and any other materials used in the review.	23

From: Page MJ, McKenzie JE, Bossuyt PM, Boutron I, Hoffmann TC, Mulrow CD, et al. The PRISMA 2020 statement: an updated guideline for reporting systematic reviews. BMJ 2021;372:n71. doi: 10.1136/bmj.n71

For more information, visit: http://www.prisma-statement.org/

Table S2. Direct-indirect estimates.

	Pain intensity outcome			Functional disability outcome	
	Difference direct-indirect estimates	Z-score and p-value[a]		Difference direct-indirect estimates	Z-score and p-value[a]
CT vs CTR	1.685±18.566, 95%CI (-34.703, 38.074)	Z=0.091, p=**0.928**	CT vs IN	16.433±11.024, 95%CI (-5.174, 38.04)	Z=1.491, p=**0.136**
CT vs IN	-5.305±18.644, 95%CI (-41.847, 31.237)	Z=-0.285, p=**0.776**	CT vs SC	-16.433±11.024, 95%CI (-38.04, 5.174)	Z=-1.491, p=**0.136**
CT vs SC	2.539±15.645, 95%CI (-28.126, 33.203)	Z=0.162, p=**0.871**	CTH vs IN	-16.433±11.024, 95%CI (-38.04, 5.174)	Z=-1.491, p=**0.136**
CTH vs CTR	-2.792±20, 95%CI (-41.991, 36.406)	Z=-0.14, p=**0.889**	CTH vs SC	16.433±11.024, 95%CI (-5.174, 38.04)	Z=1.491, p=**0.136**
CTH vs IN	5.305±18.644, 95%CI (-31.237, 41.847)	Z=0.285, p=**0.776**	CTH vs ST	-32.816±22.015, 95%CI (-75.964, 10.333)	Z=-1.491, p=**0.136**
CTH vs LST	-3.858±22.419, 95%CI (-47.798, 40.082)	Z=-0.172, p=**0.863**	IN vs ST	32.667±21.915, 95%CI (-10.286, 75.62)	Z=1.491, p=**0.136**
CTH vs SC	-1.801±18.111, 95%CI (-37.297, 33.696)	Z=-0.099, p=**0.921**			
CTH vs ST	55.565±35.392, 95%CI (-13.802, 124.931)	Z=1.57, p=**0.116**			
FEP vs CTR	-0.457±36.2, 95%CI (-71.408, 70.494)	Z=-0.013, p=**0.99**			
LST vs CTR	0.348±22.404, 95%CI (-43.563, 44.26)	Z=0.016, p=**0.988**			
SC vs CTR	-0.229±18.095, 95%CI (-35.694, 35.237)	Z=-0.013, p=**0.99**			
SC + CT vs CTR	-0.458±36.209, 95%CI (-71.426, 70.51)	Z=-0.013, p=**0.99**			
FEP vs SC	0.457±36.178, 95%CI (-70.45, 71.364)	Z=0.013, p=**0.99**			
FEP + CT vs LST	4.595±40.583, 95%CI (-74.947, 84.137)	Z=0.113, p=**0.91**			
FEP + CT vs SC	-4.595±40.576, 95%CI (-84.123, 74.934)	Z=-0.113, p=**0.91**			

IN vs ST	-55.551±35.383, 95%CI (-124.901, 13.798)	Z=-1.57, p=**0.116**
LST vs SC	-2.298±20.293, 95%CI (-42.072, 37.476)	Z=-0.113, p=**0.91**
SC vs SC + CT	-0.457±36.186, 95%CI (-71.381, 70.467)	Z=-0.013, p=**0.99**

AR: Aquatic running; AT: Aerobic training; CT: Cognitive therapy; CTH: Complementary therapy; CTR: Control; FEP: Functional exercise program; IN: Health information; LST: Lumbar stabilisation training; RT: Resistance training; SC: Standard care; ST: Stretching.

[a]Significant differences (if $p<0.05$) are represented with [a].

Table S3. Contribution studies table.

AR + FEP vs FEP	AT vs CTR	AT vs RT	CT vs CTR	CT vs IN	CT vs SC	CTH vs CTR	CTH vs IN	CTH vs LST	CTH vs SC	CTH vs ST	CTR vs FEP	CTR vs LST	CTR vs RT	CTR vs SC	CTR vs SC + CT	FEP vs SC	FEP vs SC + CT	FEP + CT vs LST	FEP + CT vs SC	IN vs ST	LST vs SC	SC vs SC + CT	FEP vs IN
Pain intensity outcome																							
0.246	0.177	0.069	0.026	0.002	0.028	0.014	0.002	0.001	0.013	<0.001	0.13	0.014	0.069	0.022	0.041	0.064	0.053	0.004	0.004	<0.001	0.009	0.012	
0.287	<0.001	<0.001	0.088	0.027	0.172	0.016	0.023	0.005	0.006	0.004	0.099	0.008	<0.001	0.01	0.022	0.129	0.058	0.001	0.001	0.004	0.002	0.036	
0.264	<0.001	<0.001	0.02	0.049	0.029	0.079	0.042	0.038	0.099	0.007	0.101	0.021	<0.001	0.006	0.024	0.109	0.054	0.005	0.005	0.007	0.012	0.03	
0.371	<0.001	<0.001	0.034	0.003	0.037	0.019	0.002	0.001	0.017	<0.001	0.212	0.018	<0.001	0.031	0.058	0.086	0.074	0.005	0.005	<0.001	0.012	0.016	
1[a]	<0.001	<0.001	<0.001	<0.001	<0.001	<0.001	<0.001	<0.001	<0.001	<0.001	<0.001	<0.001	<0.001	<0.001	<0.001	<0.001	<0.001	<0.001	<0.001	<0.001	<0.001	<0.001	
0.26	<0.001	<0.001	0.017	0.004	0.013	0.016	0.003	0.021	0.001	0.001	0.077	0.048	<0.001	0.014	0.017	0.128	0.055	0.096	0.164	0.001	0.027	0.037	
0.228	<0.001	<0.001	0.046	0.132	0.086	0.038	0.082	0.018	0.04	0.014	0.084	0.013	<0.001	0.007	0.02	0.096	0.048	0.002	0.002	0.014	0.004	0.028	
0.276	<0.001	<0.001	0.008	0.011	0.003	0.02	0.01	0.051	0.019	0.002	0.108	0.101	<0.001	0.005	0.026	0.112	0.056	0.034	0.034	0.002	0.09	0.03	

0.246	0.069	0.069	0.026	0.002	0.028	0.014	0.002	0.001	0.013	<0.001	0.13	0.014	0.177	0.022	0.041	0.064	0.053	0.004	0.004	<0.001	0.009	0.012
0.371	<0.001	<0.001	0.034	0.003	0.037	0.019	0.002	0.001	0.017	<0.001	0.086	0.018	<0.001	0.031	0.016	0.212	0.074	0.005	0.005	<0.001	0.012	0.058
0.417	<0.001	<0.001	<0.001	<0.001	<0.001	<0.001	<0.001	<0.001	<0.001	<0.001	0.083	<0.001	<0.001	<0.001	0.083	0.083	0.25	<0.001	<0.001	<0.001	<0.001	0.083
0.208	<0.001	<0.001	0.029	0.079	0.05	0.048	0.013	0.024	0.057	0.116	0.078	0.015	<0.001	0.005	0.019	0.085	0.045	0.003	0.003	0.092	0.007	0.025
<0.001	0.242	0.087	0.187	0.037	0.104	0.038	0.032	0.007	0.008	0.005	0.017	0.027	0.087	0.043	0.017	0.017	<0.001	0.006	0.006	0.005	0.014	0.017
<0.001	0.237	0.086	0.056	0.056	<0.001	0.148	0.048	0.051	0.068	0.008	0.015	0.051	0.086	0.038	0.015	0.015	<0.001	<0.001	<0.001	0.008	<0.001	0.015
<0.001	0.667	0.167	<0.001	<0.001	<0.001	<0.001	<0.001	<0.001	<0.001	<0.001	<0.001	<0.001	0.167	<0.001	<0.001	<0.001	<0.001	<0.001	<0.001	<0.001	<0.001	<0.001
<0.001	0.248	0.088	0.032	0.003	0.034	0.018	0.002	0.001	0.016	<0.001	0.188	0.017	0.088	0.028	0.053	0.08	0.068	0.005	0.005	<0.001	0.011	0.015
<0.001	0.188	0.073	0.054	0.002	0.052	0.039	0.001	0.026	0.014	<0.001	0.018	0.086	0.073	0.047	0.018	0.018	<0.001	0.126	0.135	<0.001	0.014	0.018
<0.001	0.189	0.073	0.103	0.145	0.043	0.073	0.101	0.023	0.021	0.016	0.012	0.031	0.073	0.031	0.012	0.012	<0.001	0.002	0.002	0.016	0.006	0.012

<0.001	0.242	0.087	0.04	0.01	0.03	0.051	0.009	0.064	0.002	0.002	0.013	0.179	0.087	0.033	0.013	0.013	<0.001	0.025	0.025	0.002	0.062	0.013
<0.001	0.167	0.667	<0.001	<0.001	<0.001	<0.001	<0.001	<0.001	<0.001	<0.001	<0.001	<0.001	0.167	<0.001	<0.001	<0.001	<0.001	<0.001	<0.001	<0.001	<0.001	<0.001
<0.001	0.238	0.087	0.087	0.006	0.093	0.047	0.005	0.002	0.042	0.001	0.03	0.044	0.087	0.087	0.03	0.03	<0.001	0.012	0.012	0.001	0.031	0.03
<0.001	0.248	0.088	0.032	0.003	0.034	0.018	0.002	0.001	0.016	<0.001	0.053	0.017	0.088	0.028	0.188	0.015	0.068	0.005	0.005	<0.001	0.011	0.08
<0.001	0.171	0.068	0.068	0.086	0.018	0.086	0.018	0.03	0.036	0.135	0.011	0.034	0.068	0.028	0.011	0.011	<0.001	0.001	0.001	0.104	0.003	0.011
<0.001	<0.001	<0.001	0.081	0.196	0.181	0.078	0.173	0.04	0.143	0.024	0.002	0.012	<0.001	0.005	0.002	0.002	<0.001	0.008	0.008	0.024	0.02	0.002
<0.001	<0.001	<0.001	0.421	0.051	0.157	0.053	0.044	0.009	0.011	0.007	0.024	0.037	<0.001	0.071	0.024	0.024	<0.001	0.008	0.008	0.007	0.02	0.024
<0.001	<0.001	<0.001	0.129	0.033	0.25	0.019	0.029	0.006	0.008	0.005	0.143	0.01	<0.001	0.014	0.029	0.193	0.077	0.001	0.001	0.005	0.002	0.048
<0.001	<0.001	<0.001	0.059	0.043	0.299	<0.001	0.037	0.037	0.007	0.006	0.002	0.051	<0.001	0.005	0.002	0.002	<0.001	0.129	0.272	0.006	0.042	0.002
<0.001	<0.001	<0.001	0.026	0.712	0.059	0.025	0.073	0.015	0.045	0.012	0.001	0.004	<0.001	0.002	0.001	0.001	<0.001	0.003	0.003	0.012	0.007	0.001

<0.001	<0.001	<0.001	0.114	0.072	0.231	<0.001	0.062	0.088	0.016	0.01	0.003	0.127	<0.001	0.008	0.003	0.003	<0.001	0.051	0.051	0.01	0.152	0.003
<0.001	0.087	0.087	0.187	0.037	0.104	0.038	0.032	0.007	0.008	0.005	0.017	0.027	0.242	0.043	0.017	0.017	<0.001	0.006	0.006	0.005	0.014	0.017
<0.001	<0.001	<0.001	0.054	0.04	0.728	<0.001	0.035	0.007	0.034	0.005	0.009	0.009	<0.001	0.026	0.009	0.009	<0.001	0.004	0.004	0.005	0.012	0.009
<0.001	<0.001	<0.001	0.129	0.033	0.25	0.019	0.029	0.006	0.008	0.005	0.029	0.01	<0.001	0.014	0.143	0.048	0.077	0.001	0.001	0.005	0.002	0.193
<0.001	<0.001	<0.001	0.041	0.273	0.093	0.04	0.018	0.023	0.071	0.151	0.001	0.007	<0.001	0.003	0.001	0.001	<0.001	0.005	0.005	0.255	0.011	0.001
<0.001	<0.001	<0.001	0.08	0.079	0.001	0.334	0.068	0.084	0.104	0.011	0.021	0.083	<0.001	0.062	0.021	0.021	<0.001	<0.001	<0.001	0.011	<0.001	0.021
<0.001	<0.001	<0.001	0.025	0.06	0.036	0.116	0.052	0.049	0.144	0.009	0.144	0.027	<0.001	0.007	0.031	0.155	0.07	0.006	0.006	0.009	0.016	0.039
<0.001	<0.001	<0.001	0.002	0.055	0.053	0.053	0.047	0.14	0.152	0.008	0.004	0.037	<0.001	0.01	0.004	0.004	<0.001	0.196	0.204	0.008	0.019	0.004
<0.001	<0.001	<0.001	0.028	0.091	0.063	0.027	0.584	0.015	0.048	0.06	0.001	0.005	<0.001	0.002	0.001	0.001	<0.001	0.003	0.003	0.06	0.008	0.001
<0.001	<0.001	<0.001	0.014	0.044	0.03	0.084	0.038	0.402	0.105	0.006	0.001	0.1	<0.001	0.002	0.001	0.001	<0.001	0.034	0.034	0.006	0.098	0.001

<0.001	0.086	0.086	0.056	0.056	<0.001	0.148	0.048	0.051	0.068	0.008	0.015	0.051	0.237	0.038	0.015	0.015	<0.001	<0.001	<0.001	0.008	<0.001	0.015
<0.001	<0.001	<0.001	0.012	0.09	0.101	0.067	0.078	0.07	0.391	0.012	0.012	0.002	<0.001	0.035	0.012	0.012	<0.001	0.017	0.017	0.012	0.051	0.012
<0.001	<0.001	<0.001	0.025	0.06	0.036	0.116	0.052	0.049	0.144	0.009	0.031	0.027	<0.001	0.007	0.144	0.039	0.07	0.006	0.006	0.009	0.016	0.155
<0.001	<0.001	<0.001	0.01	0.034	0.024	0.01	0.146	0.006	0.018	0.561	<0.001	0.002	<0.001	0.001	<0.001	<0.001	<0.001	0.001	0.001	0.18	0.003	<0.001
<0.001	<0.001	<0.001	0.045	0.003	0.048	0.024	0.003	0.001	0.022	<0.001	0.424	0.023	<0.001	0.046	0.087	0.118	0.107	0.006	0.006	<0.001	0.016	0.02
<0.001	<0.001	<0.001	0.076	0.002	0.074	0.055	0.002	0.037	0.02	<0.001	0.025	0.14	<0.001	0.076	0.025	0.025	<0.001	0.196	0.201	<0.001	0.019	0.025
<0.001	<0.001	<0.001	0.168	0.227	0.059	0.117	0.156	0.032	0.029	0.023	0.017	0.043	<0.001	0.044	0.016	0.017	<0.001	0.003	0.003	0.023	0.008	0.016
<0.001	<0.001	<0.001	0.055	0.014	0.042	0.083	0.012	0.1	0.003	0.002	0.018	0.402	<0.001	0.053	0.018	0.018	<0.001	0.034	0.034	0.002	0.094	0.018
<0.001	0.167	0.167	<0.001	<0.001	<0.001	<0.001	<0.001	<0.001	<0.001	<0.001	<0.001	<0.001	0.667	<0.001	<0.001	<0.001	<0.001	<0.001	<0.001	<0.001	<0.001	<0.001
<0.001	<0.001	<0.001	0.142	0.008	0.15	0.075	0.007	0.002	0.069	0.001	0.049	0.07	<0.001	0.195	0.049	0.049	<0.001	0.017	0.017	0.001	0.051	0.049

<0.001	<0.001	<0.001	0.045	0.003	0.048	0.024	0.003	0.001	0.022	<0.001	0.087	0.023	<0.001	0.046	0.424	0.02	0.107	0.006	0.006	<0.001	0.016	0.118
<0.001	<0.001	<0.001	0.098	0.12	0.023	0.139	0.024	0.043	0.05	0.207	0.015	0.048	<0.001	0.038	0.015	0.015	<0.001	0.001	0.001	0.145	0.003	0.015
<0.001	<0.001	<0.001	0.02	0.004	0.016	0.02	0.004	0.026	0.001	0.001	0.1	0.063	<0.001	0.018	0.021	0.19	0.07	0.124	0.236	0.001	0.035	0.049
<0.001	<0.001	<0.001	0.061	0.173	0.112	0.05	0.107	0.022	0.052	0.017	0.11	0.016	<0.001	0.008	0.025	0.127	0.06	0.002	0.002	0.017	0.004	0.035
<0.001	<0.001	<0.001	0.01	0.014	0.004	0.026	0.012	0.065	0.025	0.002	0.156	0.148	<0.001	0.006	0.034	0.161	0.073	0.045	0.045	0.002	0.132	0.04
<0.001	0.088	0.088	0.032	0.003	0.034	0.018	0.002	0.001	0.016	<0.001	0.188	0.017	0.248	0.028	0.053	0.08	0.068	0.005	0.005	<0.001	0.011	0.015
<0.001	<0.001	<0.001	0.045	0.003	0.048	0.024	0.003	0.001	0.022	<0.001	0.118	0.023	<0.001	0.046	0.02	0.424	0.107	0.006	0.006	<0.001	0.016	0.087
<0.001	<0.001	<0.001	<0.001	<0.001	<0.001	<0.001	<0.001	<0.001	<0.001	<0.001	0.125	<0.001	<0.001	<0.001	0.125	0.125	0.5	<0.001	<0.001	<0.001	<0.001	0.125
<0.001	<0.001	<0.001	0.036	0.097	0.061	0.063	0.016	0.029	0.074	0.151	0.1	0.018	<0.001	0.007	0.024	0.109	0.054	0.003	0.003	0.114	0.008	0.031
<0.001	<0.001	<0.001	0.024	0.163	0.139	0.019	0.121	0.069	0.052	0.019	0.002	0.033	<0.001	0.006	0.002	0.002	<0.001	0.126	0.177	0.019	0.025	0.002

<0.001	<0.001	<0.001	0.011	0.009	0.02	<0.001	0.007	0.034	0.025	0.001	0.005	0.033	<0.001	0.013	0.005	0.005	<0.001	0.586	0.153	0.001	0.086	0.005
<0.001	0.073	0.073	0.054	0.002	0.052	0.039	0.001	0.026	0.014	<0.001	0.018	0.086	0.188	0.047	0.018	0.018	<0.001	0.126	0.135	<0.001	0.014	0.018
<0.001	<0.001	<0.001	0.011	0.009	0.02	<0.001	0.007	0.034	0.025	0.001	0.005	0.033	<0.001	0.013	0.005	0.005	<0.001	0.153	0.586	0.001	0.086	0.005
<0.001	<0.001	<0.001	0.02	0.004	0.016	0.02	0.004	0.026	0.001	0.001	0.021	0.063	<0.001	0.018	0.1	0.049	0.07	0.124	0.236	0.001	0.035	0.19
<0.001	<0.001	<0.001	0.01	0.091	0.08	0.029	0.023	0.079	0.075	0.161	0.003	0.028	<0.001	0.006	0.003	0.003	<0.001	0.126	0.148	0.113	0.018	0.003
<0.001	<0.001	<0.001	0.048	0.151	0.103	0.026	0.186	0.159	0.027	0.026	0.001	0.08	<0.001	0.004	0.001	0.001	<0.001	0.034	0.034	0.026	0.09	0.001
<0.001	0.073	0.073	0.103	0.145	0.043	0.073	0.101	0.023	0.021	0.016	0.012	0.031	0.189	0.031	0.012	0.012	<0.001	0.002	0.002	0.016	0.006	0.012
<0.001	<0.001	<0.001	0.015	0.277	0.262	0.023	0.145	0.028	0.116	0.021	0.008	0.003	<0.001	0.02	0.008	0.008	<0.001	0.008	0.008	0.021	0.022	0.008
<0.001	<0.001	<0.001	0.061	0.173	0.112	0.05	0.107	0.022	0.052	0.017	0.025	0.016	<0.001	0.008	0.11	0.035	0.06	0.002	0.002	0.017	0.004	0.127
<0.001	<0.001	<0.001	0.01	0.035	0.024	0.01	0.146	0.006	0.018	0.18	<0.001	0.002	<0.001	0.001	<0.001	<0.001	<0.001	0.001	0.001	0.56	0.003	<0.001

<0.001	0.087	0.087	0.04	0.01	0.03	0.051	0.009	0.064	0.002	0.002	0.013	0.179	0.242	0.033	0.013	0.013	<0.001	0.025	0.025	0.002	0.062	0.013
<0.001	<0.001	<0.001	0.03	0.022	0.052	<0.001	0.019	0.098	0.076	0.003	0.013	0.093	<0.001	0.038	0.013	0.013	<0.001	0.086	0.086	0.003	0.343	0.013
<0.001	<0.001	<0.001	0.01	0.014	0.004	0.026	0.012	0.065	0.025	0.002	0.034	0.148	<0.001	0.006	0.156	0.04	0.073	0.045	0.045	0.002	0.132	0.161
<0.001	<0.001	<0.001	0.025	0.08	0.055	0.039	0.039	0.172	0.048	0.221	0.001	0.068	<0.001	0.002	0.001	0.001	<0.001	0.028	0.028	0.119	0.071	0.001
<0.001	0.087	0.087	0.087	0.006	0.093	0.047	0.005	0.002	0.042	0.001	0.03	0.044	0.238	0.087	0.03	0.03	<0.001	0.012	0.012	0.001	0.031	0.03
<0.001	0.088	0.088	0.032	0.003	0.034	0.018	0.002	0.001	0.016	<0.001	0.053	0.017	0.248	0.028	0.188	0.015	0.068	0.005	0.005	<0.001	0.011	0.08
<0.001	0.068	0.068	0.068	0.086	0.018	0.086	0.018	0.03	0.036	0.135	0.011	0.034	0.171	0.028	0.011	0.011	<0.001	0.001	0.001	0.104	0.003	0.011
<0.001	<0.001	<0.001	0.045	0.003	0.048	0.024	0.003	0.001	0.022	<0.001	0.02	0.023	<0.001	0.046	0.118	0.087	0.107	0.006	0.006	<0.001	0.016	0.424
<0.001	<0.001	<0.001	0.002	0.142	0.14	0.035	0.017	0.038	0.154	0.211	0.008	<0.001	<0.001	0.021	0.008	0.008	<0.001	0.01	0.01	0.159	0.028	0.008
<0.001	<0.001	<0.001	0.036	0.097	0.061	0.063	0.016	0.029	0.074	0.151	0.024	0.018	<0.001	0.007	0.1	0.031	0.054	0.003	0.003	0.114	0.008	0.109

Functional disability outcome

0.172	0.121	0.051	0.172	0.135	0.038	0.026	0.038	0.012	0.051	0.012	0.172
0.292	<0.001	<0.001	<0.001	0.238	0.054	0.038	0.054	0.016	<0.001	0.016	0.292
0.275	<0.001	<0.001	<0.001	0.057	0.057	0.158	0.057	0.06	<0.001	0.06	0.275
0.224	<0.001	<0.001	0.224	0.178	0.046	0.032	0.046	0.014	<0.001	0.014	0.224
1[a]	<0.001	<0.001	<0.001	<0.001	<0.001	<0.001	<0.001	<0.001	<0.001	<0.001	<0.001
0.5	<0.001	<0.001	<0.001	<0.001	<0.001	<0.001	<0.001	<0.001	<0.001	<0.001	0.5
0.172	0.052	0.052	0.172	0.135	0.038	0.026	0.038	0.012	0.121	0.012	0.172
0.243	<0.001	<0.001	<0.001	0.152	0.152	0.065	0.091	0.026	<0.001	0.026	0.243
0.29	<0.001	<0.001	<0.001	0.024	0.024	0.06	0.024	0.084	<0.001	0.206	0.29
<0.001	0.332	0.112	0.444	<0.001	<0.001	<0.001	<0.001	<0.001	0.112	<0.001	<0.001
<0.001	0.161	0.065	0.227	0.093	0.134	0.066	0.134	0.027	0.065	0.027	<0.001

<0.001	0.665	0.168	<0.001	<0.001	<0.001	<0.001	<0.001	<0.001	0.168	<0.001	<0.001
<0.001	0.149	0.061	0.21	0.166	0.043	0.03	0.043	0.013	0.061	0.013	0.21
<0.001	0.194	0.075	0.269	0.218	0.051	0.036	0.051	0.015	0.075	0.015	<0.001
<0.001	0.164	0.672	<0.001	<0.001	<0.001	<0.001	<0.001	<0.001	0.164	<0.001	<0.001
<0.001	0.212	0.081	0.292	0.019	0.274	0.013	0.019	0.006	0.081	0.006	<0.001
<0.001	0.15	0.062	0.212	0.131	0.081	0.009	0.081	0.09	0.062	0.122	<0.001
<0.001	<0.001	<0.001	<0.001	0.19	0.286	0.142	0.286	0.048	<0.001	0.048	<0.001
<0.001	<0.001	<0.001	1[a]	<0.001	<0.001	<0.001	<0.001	<0.001	<0.001	<0.001	<0.001
<0.001	<0.001	<0.001	<0.001	0.356	0.067	0.048	0.067	0.019	<0.001	0.019	0.423
<0.001	<0.001	<0.001	<0.001	0.713	0.088	0.064	0.088	0.024	<0.001	0.024	<0.001

<0.001	0.112	0.112	0.444	<0.001	<0.001	<0.001	<0.001	<0.001	0.332	<0.001	<0.001
<0.001	<0.001	<0.001	<0.001	0.032	0.896[a]	0.023	0.032	0.009	<0.001	0.009	<0.001
<0.001	<0.001	<0.001	<0.001	0.277	0.143	0.016	0.143	0.159	<0.001	0.261	<0.001
<0.001	<0.001	<0.001	0.321	0.131	0.191	0.095	0.191	0.036	<0.001	0.036	<0.001
<0.001	<0.001	<0.001	<0.001	0.071	0.071	0.238	0.071	0.08	<0.001	0.08	0.389
<0.001	<0.001	<0.001	<0.001	0.095	0.095	0.475	0.095	0.12	<0.001	0.12	<0.001
<0.001	0.066	0.066	0.227	0.093	0.133	0.066	0.133	0.027	0.161	0.027	<0.001
<0.001	<0.001	<0.001	<0.001	0.099	0.099	0.072	0.677	0.027	<0.001	0.027	<0.001
<0.001	<0.001	<0.001	<0.001	0.036	0.036	0.118	0.036	0.621	<0.001	0.154	<0.001
<0.001	<0.001	<0.001	0.292	0.238	0.054	0.038	0.054	0.016	<0.001	0.016	0.292

<0.001	<0.001	<0.001	0.423	0.356	0.067	0.048	0.067	0.019	<0.001	0.019	<0.001
<0.001	0.168	0.168	<0.001	<0.001	<0.001	<0.001	<0.001	<0.001	0.664	<0.001	<0.001
<0.001	<0.001	<0.001	0.472	0.024	0.448	0.017	0.024	0.007	<0.001	0.007	<0.001
<0.001	<0.001	<0.001	0.293	0.186	0.108	0.012	0.108	0.12	<0.001	0.174	<0.001
<0.001	<0.001	<0.001	<0.001	<0.001	<0.001	<0.001	<0.001	<0.001	<0.001	<0.001	1[a]
<0.001	0.061	0.061	0.21	0.166	0.043	0.03	0.043	0.013	0.149	0.013	0.21
<0.001	<0.001	<0.001	<0.001	0.203	0.203	0.087	0.12	0.033	<0.001	0.033	0.322
<0.001	<0.001	<0.001	<0.001	0.029	0.029	0.08	0.029	0.108	<0.001	0.309	0.417
<0.001	0.075	0.075	0.269	0.218	0.051	0.036	0.051	0.015	0.194	0.015	<0.001
<0.001	<0.001	<0.001	<0.001	0.304	0.304	0.13	0.174	0.044	<0.001	0.044	<0.001

	<0.001	<0.001	<0.001	<0.001	0.036	0.036		0.119		0.036	0.155			<0.001							0.618			<0.001
	<0.001	0.081	0.081	0.292	0.019	0.273		0.013		0.019	0.006			0.211							0.006			<0.001
	<0.001	0.062	0.062	0.212	0.131	0.081		0.009		0.081	0.09			0.15							0.122			<0.001
	<0.001	<0.001	<0.001	<0.001	0.155	0.155		0.007		0.264	0.257			<0.001							0.162			<0.001

AR: Aquatic running; AT: Aerobic training; CT: Cognitive therapy; CTH: Complementary therapy; CTR: Control; FEP: Functional exercise program; IN: Health information; LST: Lumbar stabilisation training; RT: Resistance training; SC: Standard care; ST: Stretching.

Values higher 0.8 are represented with [a].

Table S4. Characteristics of studies.

Pain intensity outcome				Functional disability outcome			
**Study**	**Comparison**	**Number of participants**	**Effect size ± Standard Error**	**Study**	**Comparison**	**Number of participants**	**Effect size ± Standard Error**
Amorim, 2019	CT vs IN	68	-0.4±0.538	Amorim, 2019	CT vs IN	68	1.5±0.84
Bendix, 1998	FEP vs CTR	139	-0.562±0.408	Cuesta-Vargas, 2011	FEP vs AR + FEP	49	-34.4±3.56
	FEP vs SC	118	-0.77±0.539	Fersum, 2013	CT vs SC	94	2.3±0.973
	FEP vs SC + CT	124	-0.77±0.52	Gibbs, 2018	CT vs SC	27	8.8±3.501
Cuesta-Vargas, 2011	FEP vs AR + FEP	49	-1.9±4.638	Kell, 2009	AT vs CTR	18	-16.1±1.043
Fersum, 2013	CT vs SC	94	-1.7±0.304		AT vs RT	18	-12.3±0.852
Gibbs, 2018	CT vs SC	27	-1.3±0.552		RT vs CTR	18	-3.8±1.066
Járomi, 2018	CT vs IN	137	-41.56±0.99	Krein, 2013	CT vs SC	229	2.035±0.635
Kell, 2009	AT vs CTR	18	-0.2±0.268	Phattharasupharek, 2019	FEP vs IN	72	-36.1±2.488
	AT vs RT	18	1.8±0.301	Sherman, 2011	CTH vs IN	137	-2.77±0.691
	RT vs CTR	18	-2±0.277		CTH vs ST	183	-1.04±0.516
Khodadad, 2020	FEP + CT vs LST	34	0.6±0.339		ST vs IN	136	-1.73±0.651
	FEP + CT vs SC	35	-2.4±0.386	Tekur, 2012	CTH vs SC	91	16.14±2.255
	LST vs SC	35	-3±0.408	Williams, 2018	CT vs CTR	159	0.3±0.715
Krein, 2013	CT vs SC	229	-0.1±0.203				

Phattharasupharek, 2019	CTH vs IN	72	-34.4±3.493
Sherman, 2011	CTH vs IN	137	-1.14±0.312
	CTH vs ST	183	-0.73±0.231
	ST vs IN	136	-0.41±0.318
Tekur, 2012	CTH vs SC	80	-2.25±0.34
Williams, 2018	CT vs CTR	159	-0.4±0.29
Zou, 2019	CTH vs CTR	28	-2.2±0.26
	CTH vs LST	30	-0.8±0.239
	LST vs CTR	28	-1.4±0.238
	CTR vs SC	77	-0.208±0.485
	CTR vs SC + CT	83	-0.208±0.463
	SC vs SC + CT	62	0±0.582

AR: Aquatic running; AT: Aerobic training; CT: Cognitive therapy; CTH: Complementary therapy; CTR: Control; FEP: Functional exercise program; IN: Health information; LST: Lumbar stabilisation training; RT: Resistance training; SC: Standard care; ST: Stretching.

Table S5. Ranking table of interventions.

Combined pain-disability outcomes		Pain intensity outcome				Functional disability outcome			
	P-score		P-score		SUCRA		P-score		SUCRA
ST	0.836	CT	0.588	LST	0.618	FEP	0.99	FEP	0.993
RT	0.662	LST	0.582	FEP + CT	0.608	AT	0.861	AT	0.846
SC	0.543	RT	0.577	RT	0.606	SC	0.67	SC	0.647
CTR	0.521	FEP + CT	0.570	CTH	0.582	RT	0.545	RT	0.569
CTH	0.438	CTH	0.570	AT	0.556	CT	0.411	CTR	0.407
IN	0.379	FEP	0.548	CT	0.554	CTR	0.407	CT	0.398
AT	0.366	CTR	0.538	SC	0.544	AR + FEP	0.373	AR + FEP	0.358
CT	0.238	AT	0.534	SC + CT	0.533	IN	0.278	ST	0.284
FEP	0.224	SC + CT	0.527	CTR	0.531	ST	0.269	IN	0.258
AR + FEP	0.12	SC	0.526	FEP	0.508	CTH	0.195	CTH	0.241
		AR + FEP	0.500	AR + FEP	0.458	FEP + CT		FEP + CT	
		ST	0.335	ST	0.314	LST		LST	
		IN	0.104	IN	0.088	SC + CT		SC + CT	

AR: Aquatic running; AT: Aerobic training; CT: Cognitive therapy; CTH: Complementary therapy; CTR: Control; FEP: Functional exercise program; IN: Health information; LST: Lumbar stabilisation training; RT: Resistance training; SC: Standard care; ST: Stretching.

Table S6. Pairwise comparison for combined pain intensity-functional disability.

CTR - AR + FEP	51.847 (-38.332, 142.027)	AR + FEP - AT	36.718 (-22.169, 96)	AT - CT	-4.655 (-8.367, -1)	CTR - AR + FEP	51.847 (-38.332, 142.027)	CTH - FEP	-18.345 (-51.568, 15)	FEP - IN	17.332 (-15.974, 51)	IN - RT	-1.769 (-35.5, 32)	RT - ST	24.02 (-34.792, 83)	ST - SC	-10.311 (-43.899, 23)
CTR - AT	15.129 (-19.341, 49.599)	AR + FEP - CT	32.064 (-26.7, 91)	AT - CTH	4.572 (-29.096, 38)	CTR - AT	15.129 (-19.341, 49.599)	CTH - IN	-1.013 (-3.359, 1)	FEP - RT	15.562 (-18.251, 49)	IN - ST	22.25 (-12.142, 57)	RT - SC	13.708 (-20.837, 48)		
CTR - CT	19.784 (-14.677, 54.244)	AR + FEP - CTH	41.291 (-26.234, 109)	AT - FEP	-13.772 (-47.605, 20)	CTR - CT	19.784 (-14.677, 54.244)	CTH - RT	-2.782 (-36.431, 31)	FEP - ST	39.582 (-27.974, 107)	IN - SC	11.939 (-22.13, 46)				
CTR - CTH	10.557 (-23.687, 44.801)	AR + FEP - FEP	22.946 (-11.532, 57)	AT - IN	3.56 (-30.19, 37)	CTR - CTH	10.557 (-23.687, 44.801)	CTH - ST	21.238 (-13.315, 56)	FEP - SC	29.271 (-29.407, 88)						
CTR - FEP	28.901 (-29.831, 87.634)	AR + FEP - IN	40.278 (-27.369, 108)	AT - RT	1.79 (-0.694, 4)	CTR - FEP	28.901 (-29.831, 87.634)	CTH - SC	10.926 (-23.224, 45)								
CTR - IN	11.57 (-22.594, 45.733)	AR + FEP - RT	38.508 (-20.368, 97)	AT - ST	25.81 (-33.013, 85)	CTR - IN	11.57 (-22.594, 45.733)										
CTR - RT	13.339 (-21.112, 47.79)	AR + FEP - ST	62.528 (-39.38, 164)	AT - SC	15.498 (-19.065, 50)	CTR - RT	13.339 (-21.112, 47.79)										
CTR - ST	-10.681 (-44.364, 23.003)	AR + FEP - SC	52.217 (-37.927, 142)			CTR - ST	-10.681 (-44.364, 23.003)										
CTR - SC	-0.369 (-2.914, 2.175)					CTR - SC	-0.369 (-2.914, 2.175)										
						CT - CTH	9.227 (-24.236, 43)										

CT - FEP	-9.118 (-42.739, 25)
CT - IN	8.214 (-25.331, 42)
CT - RT	6.445 (2.908, 10)
CT - ST	30.465 (-28.245, 89)
CT - SC	20.153 (-14.214, 55)
CTH - FEP	-18.345 (-51.568, 15)
CTH - IN	-1.013 (-3.359, 1)
CTH - RT	-2.782 (-36.431, 31)
CTH - ST	21.238 (-13.315, 56)
CTH - SC	10.926 (-23.224, 45)

AR: Aquatic running; AT: Aerobic training; CT: Cognitive therapy; CTH: Complementary therapy; CTR: Control; FEP: Functional exercise program; IN: Health information; LST: Lumbar stabilisation training; RT: Resistance training; SC: Standard care; ST: Stretching. [a]Significant differences (if $p<0.05$) are represented with [a].

Figure S1. Covariate-weighted networks.

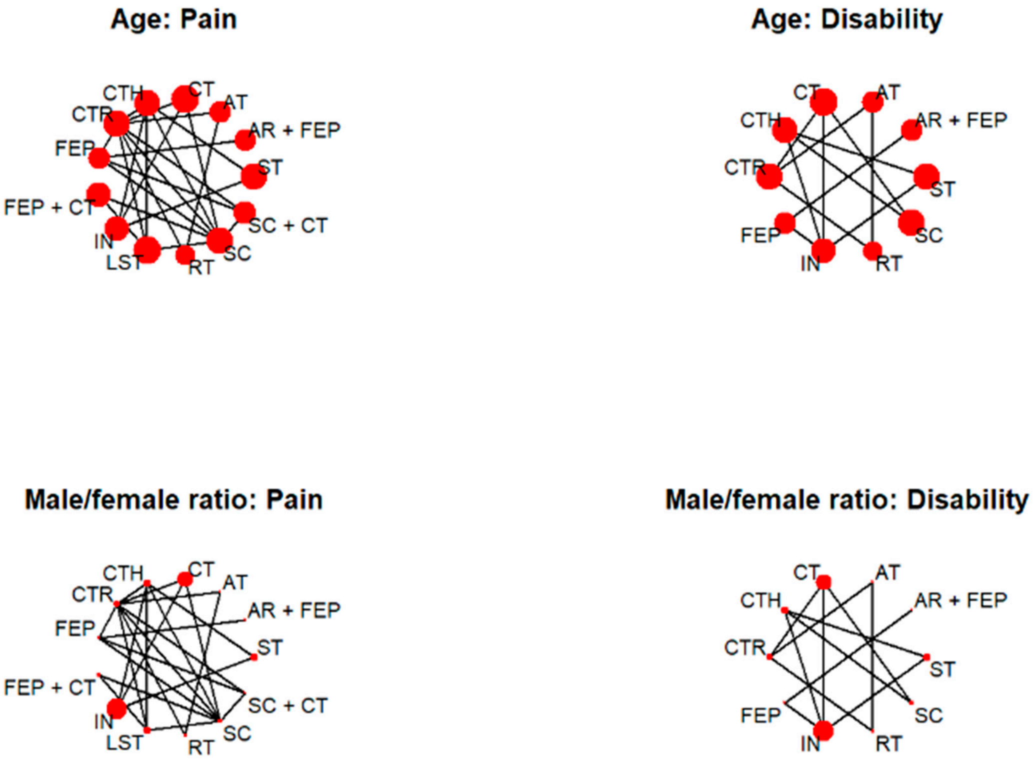

AR: Aquatic running; AT: Aerobic training; CT: Cognitive therapy; CTH: Complementary therapy; CTR: Control; FEP: Functional exercise program; IN: Health information; LST: Lumbar stabilisation training; RT: Resistance training; SC: Standard care; ST: Stretching.

Figure S2. Direct evidence proportion for each network estimate (random-effects model).

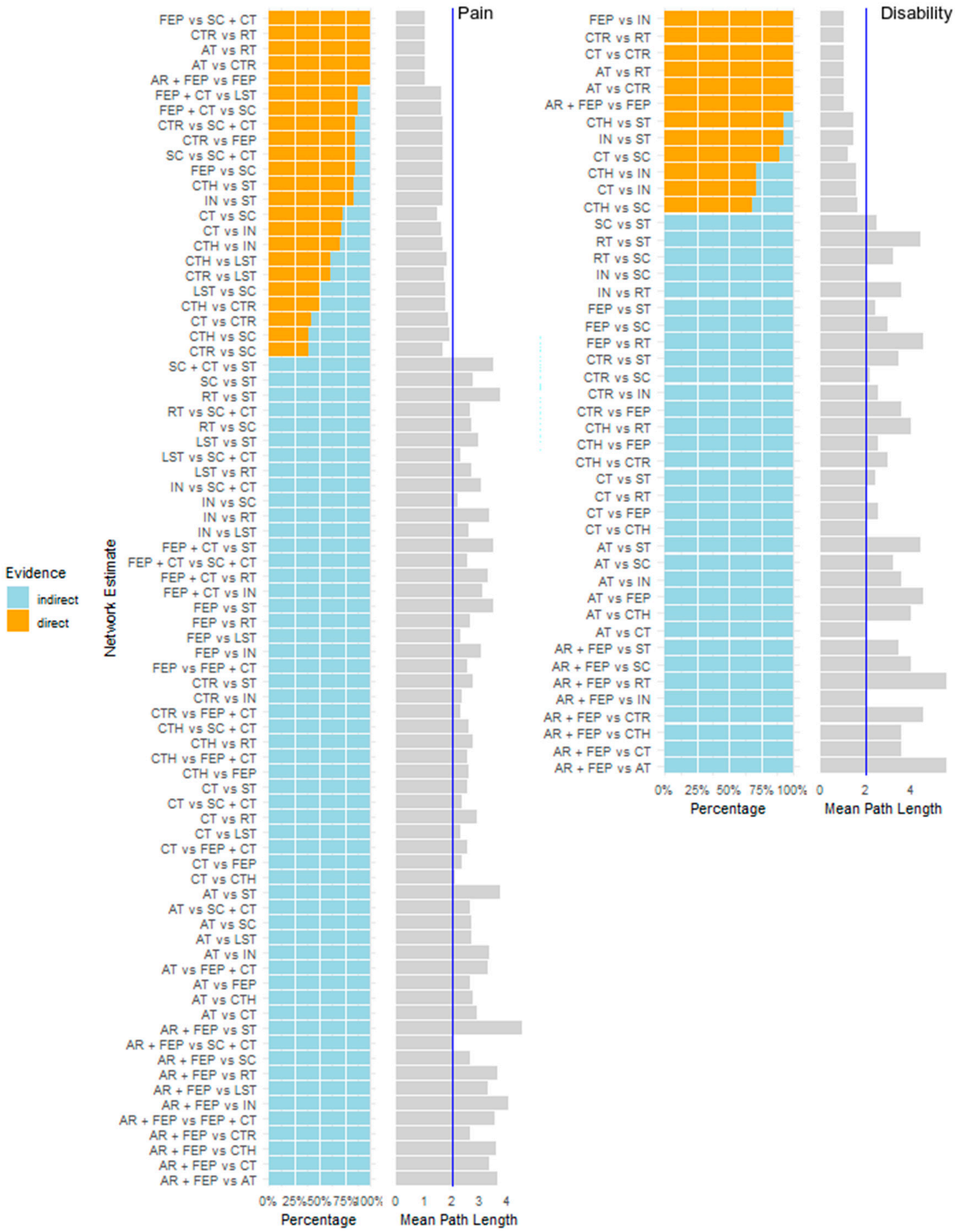

AR: Aquatic running; AT: Aerobic training; CT: Cognitive therapy; CTH: Complementary therapy; CTR: Control; FEP: Functional exercise program; IN: Health information; LST: Lumbar stabilisation training; RT: Resistance training; SC: Standard care; ST: Stretching.

Figure S3. Heterogeneity studies contribution plot.

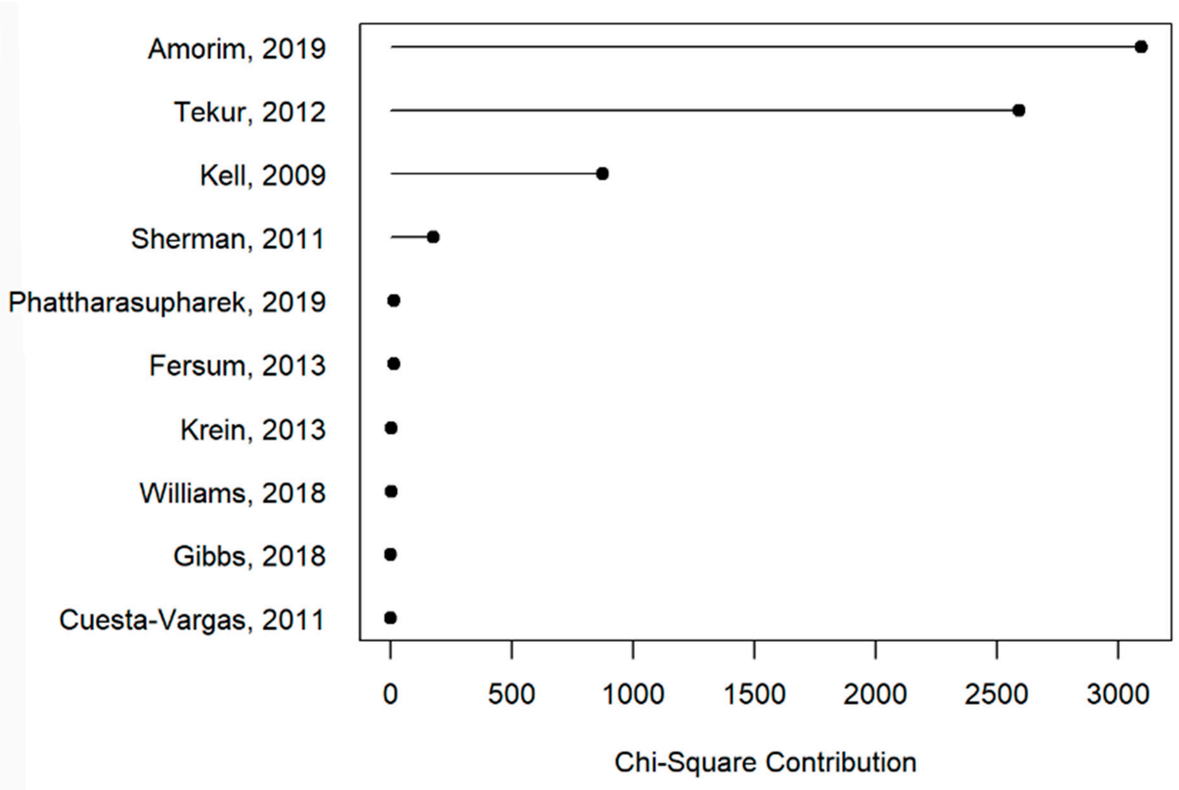

AR: Aquatic running; AT: Aerobic training; CT: Cognitive therapy; CTH: Complementary therapy; CTR: Control; FEP: Functional exercise program; IN: Health information; LST: Lumbar stabilisation training; RT: Resistance training; SC: Standard care; ST: Stretching.

Figure S4. Pain and functional disability SUCRA rankogram plots.

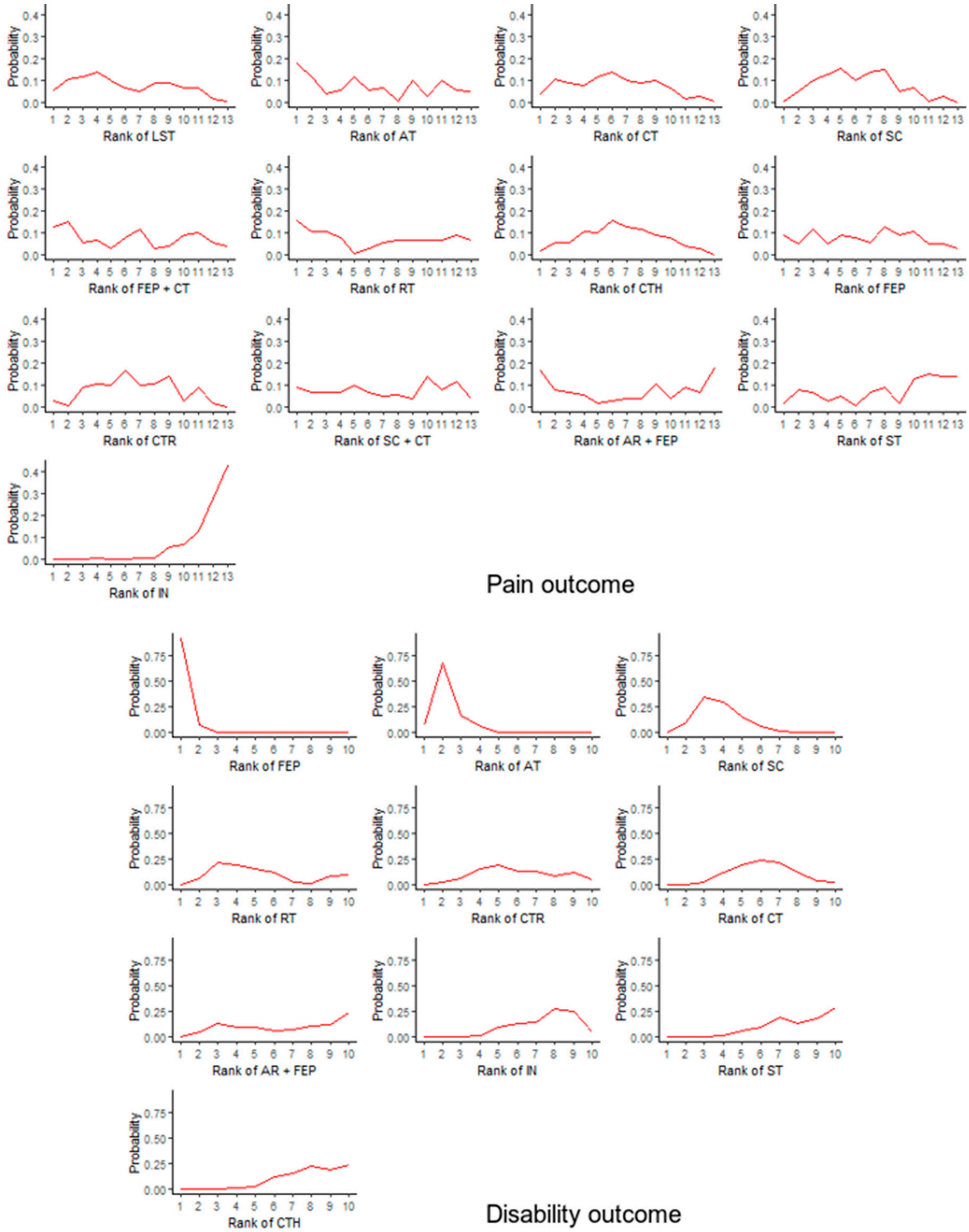

AR: Aquatic running; AT: Aerobic training; CT: Cognitive therapy; CTH: Complementary therapy; CTR: Control; FEP: Functional exercise program; IN: Health information; LST: Lumbar stabilisation training; RT: Resistance training; SC: Standard care; ST: Stretching.

Figure S5. Publication bias funnel plots.

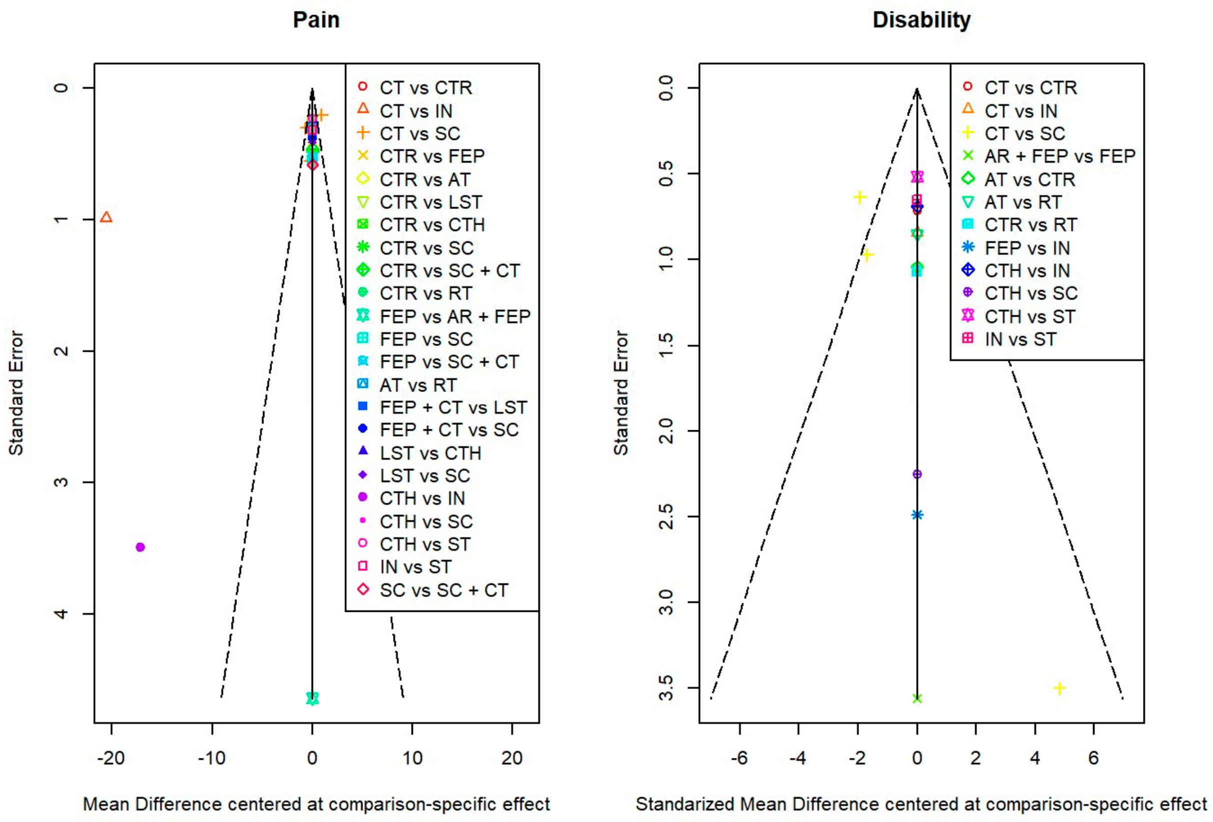

AR: Aquatic running; AT: Aerobic training; CT: Cognitive therapy; CTH: Complementary therapy; CTR: Control; FEP: Functional exercise program; IN: Health information; LST: Lumbar stabilisation training; RT: Resistance training; SC: Standard care; ST: Stretching.
